# *Ribes nigrum L*. polysaccharides as multifunctional bioactive agents: current advances, challenges, and future directions in functional food and pharmaceutical development

**DOI:** 10.3389/fnut.2026.1857689

**Published:** 2026-06-03

**Authors:** Zhe Wang, Lina Hao, Yan Gao

**Affiliations:** 1 Department of Pharmacy, Children's Hospital Affiliated to Shandong University (Jinan Children's Hospital), Jinan, China; 2 Science, Education and Foreign Affairs Section, Children's Hospital Affiliated to Shandong University (Jinan Children's Hospital), Jinan, China

**Keywords:** *Ribes nigrum L*., polysaccharides, extraction, structural features, bibliometrics, applications

## Abstract

*Ribes nigrum* L. (Blackcurrant) is a widely cultivated shrub that originated in the temperate and cold regions of the Northern Hemisphere. It has been used for its medicinal properties throughout history and is rich in various bioactive compounds, including polysaccharides. Published studies have shown that *R. nigrum* polysaccharides (RNPs) are key macromolecules with multiple health-promoting effects, such as antioxidant, anti-diabetic, immunomodulatory, anti-inflammatory, anti-tumor, anti-aging, anti-gout, etc. Although extensive research has been conducted on the extraction and purification methods, structural characteristics, and pharmacological activities of RNPs in the fruits, seeds, and other parts of the plant, knowledge regarding their structure-activity relationships, safety features, and pharmacokinetic behaviors is still limited. This is mainly due to the complex and variable structure of natural polysaccharides, which makes it difficult to precisely characterize them. This review systematically summarizes the latest progress in the extraction, purification, structural characterization, biological activities, and potential applications of RNPs, and discusses the current challenges and future prospects of using them as therapeutic drugs and functional food components. It also points out that future research should combine multiple analytical techniques (such as atomic force microscopy) to address the limitations of current studies.

## Graphical Abstract

**Figure:**
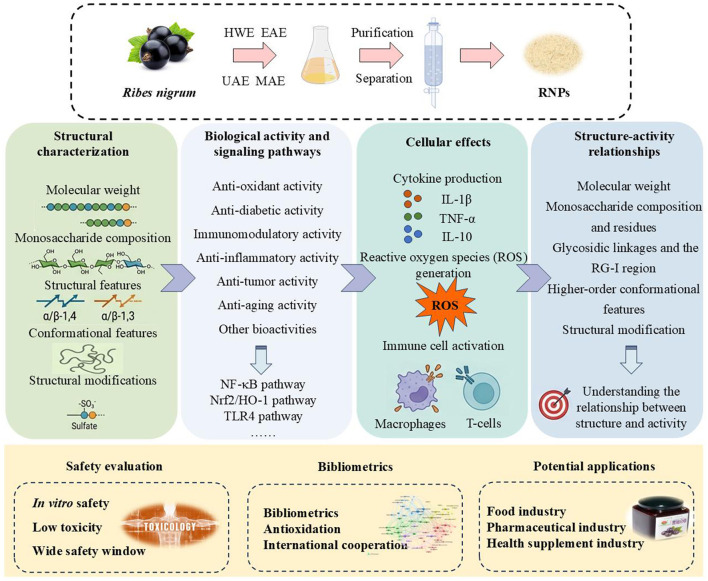

## 1 Introduction

**Figure 1:**
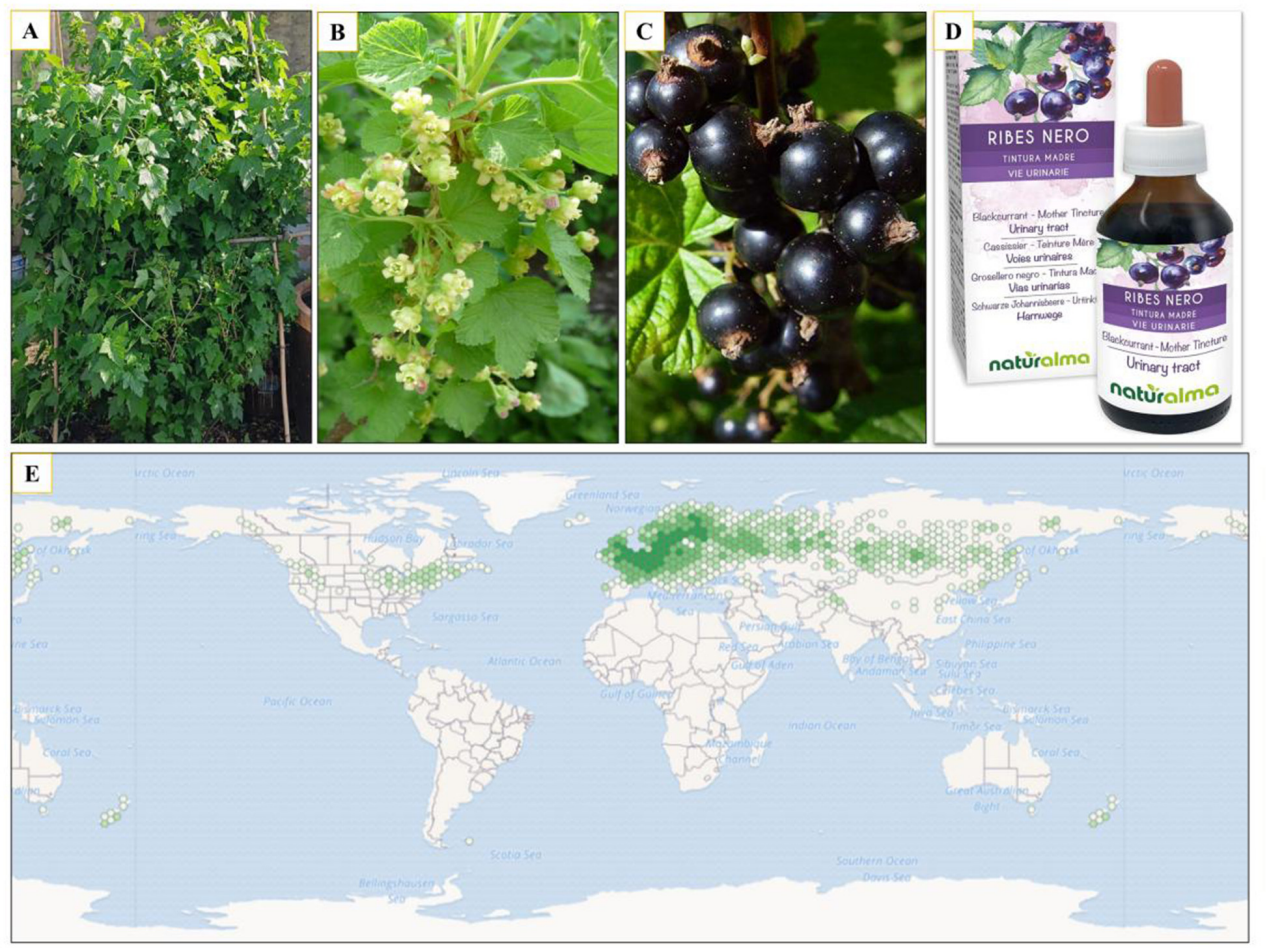
Botanical characteristics of *R. nigrum*: **(A)** whole plant; **(B)** flowers; **(C)** foods; **(D)** product; **(E)** world distribution (source: https://www.gbif.org/species/2986192).

*Ribes nigrum* L. (blackcurrant), a deciduous shrub belonging to the family *Grossulariaceae* and genus *Ribes*, is of pronounced economic and health significance (1, 2). Native to Northern Asia and Europe, this species is primarily distributed across temperate and cold regions of the Northern Hemisphere (3, 4). Its deep-purple to black berries, valued for their distinctive flavor, are internationally recognized as a “superfruit” on account of their exceptionally high concentrations of bioactive constituents—particularly anthocyanins, ascorbic acid and diverse polyphenolic metabolites (5–7). Driven by the rapid expansion of functional-food and phytopharmaceutical research, the utilization of *R. nigrum* has transcended the edible fruit; leaves and buds, traditionally employed across Europe as anti-inflammatory and anti-oxidation, have now emerged as a promising reservoir for drug discovery (8, 9). These historical therapeutic indications are being systematically validated by contemporary pharmacological investigations, thereby catalyzing intensive efforts to elucidate the molecular basis underlying the plant's bioactivities. Figure 1 concisely shows the core information of *R. nigrum*: from the whole plant morphology (A), floral characteristics (B), edible fruit (C), typical processed products (D) to its global distribution pattern (E), which provides an intuitive species background for subsequent experiments.

Modern phytochemical investigations have revealed that the health-promoting potential of *R. nigrum* is primarily attributable to the synergistic interplay of a spectrum of bioactive constituents. Among these, anthocyanins constitute the most characteristic class of metabolites in the berries; their potent anti-oxidant and anti-inflammatory properties have been extensively corroborated by both *in vitro* and *in vivo* studies, demonstrating considerable promise in mitigating oxidative stress, ameliorating metabolic syndrome, and conferring neuroprotection (10, 11). *R. nigrum* seed oil is exceptionally enriched in polyunsaturated fatty acids, particularly γ-linolenic acid, whose capacity to modulate epidermal barrier function and suppress inflammatory cascades has attracted substantial research interest (12). Additionally, *R. nigrum* leaves—often described as a repository of polyphenols—contain flavonols and proanthocyanidins at concentrations exceeding those recorded in the fruit; these compounds are regarded as the principal effectors underlying the leaf's anti-rheumatic, anti-microbial, and vasoprotective activities (13, 14). Specifically, research results show that the content of quercetin-3-O-anisol in leaves is as high as 28.60 mg/g dry weight, while it is only 1.99 mg/g dry weight in fruits. The contents of catechin, epicatechin and various kaempferol glycosides in leaves are also significantly higher than those in fruits. These quantitative data provide direct evidence that specific phenolic substances in leaves exceed those in fruits, and suggest that in the study of polysaccharides, attention should be paid to the potential interference of the phenolic background of extracts from different parts on the functional activity of polysaccharides (15). Notably, while secondary metabolites have been scrutinized in considerable detail, the polysaccharides—primary metabolites that constitute the predominant biomass fraction of *R. nigrum*—have remained relatively under-explored. Over the past decade, plant-derived polysaccharides have emerged as a frontier topic in biomedicine owing to their multifaceted immunomodulatory, anti-tumor, anti-oxidant, and gut microbiota-modulating activities (16, 17). Preliminary investigations have demonstrated that polysaccharides isolated from both the fruits and leaves of *R. nigrum* exhibit promising bioactivities, including notable antioxidant capacity and immunostimulatory effects (18, 19). Consequently, a systematic elucidation of the extraction protocols, chemical architectures, biological functionalities, and structure-activity relationships of *R. nigrum* polysaccharides (RNPs) is imperative for the comprehensive valorization of this traditional medicinal and edible plant and for the development of high-value pharmaceutical and nutraceutical applications.

Given the significant application potential of RNPs in the functional food and pharmaceutical industries, a comprehensive summary of their structural characteristics, biological mechanisms, and potential toxicity is still lacking, despite considerable research progress. Therefore, this review aims to comprehensively consolidate recent advances in the extraction, purification, structural characterization, biological activities, and safety of RNPs, and to provide an in-depth discussion of their structure-activity relationships. It is anticipated that this work will offer a theoretical foundation and future perspectives for the further development and application of RNPs.

## 2 Botany and traditional background

*R. nigrum* is a deciduous small shrub, with a plant height of up to 1 to 2 m (20). Native to the temperate regions of Europe and northern Asia, it is commercially cultivated in China mainly in the Altai Mountains of Xinjiang, the Greater Khingan Range of Heilongjiang, as well as in Jilin and Liaoning provinces (21–23). This species exhibits strong cold tolerance, prefers cool climates, and thrives under conditions of an average annual temperature of 2~15°C and annual precipitation of 500~800 mm (24, 25). As the quality and active constituent contents of its fruit are closely related to developmental status, the ripening degree of fruits serves as a key variable. Although the available evidence is limited, existing studies have shown that the fruit ripening stage can affect its quality and the quantity of its components. Therefore, when comparing the results of different studies, this factor should be taken into consideration (26).

In traditional medicine, the fruits, leaves, buds, and even bark of blackcurrant have been utilized for centuries by various ethnic groups in Europe and northern Asia (27–29). In particular, the uses of its fruits are the most extensive. Moreover, from the perspective of polysaccharides, these fruits have also been the subject of the most in-depth research. In European folk medicine, dried leaves are commonly decocted or infused as a diuretic and anti-inflammatory agent to treat rheumatoid arthralgia, gout, and urinary tract infections, and are also used as a mouthwash to relieve sore throat and bleeding gums (30, 31). The fruits, rich in vitamins, are consumed fresh or processed into juices, jams, and syrups to prevent scurvy, enhance immunity, and protect against colds and bronchitis. In Nordic countries and Russia, the fruit is additionally believed to alleviate eye fatigue and improve night vision (32–34). In Siberian folk practice, fresh juice is applied directly to eczema, minor wounds, and insect bites. Since its introduction to Northeast China and Xinjiang in the mid-20th century, the medicinal value of blackcurrant has integrated with local customs. Among the Kazakh ethnic group in the Altai region of Xinjiang, the fruits are commonly collected to make jams and beverages, which are considered nourishing and antitussive (35). In recent years, based on these traditional applications, some blackcurrant functional products have also been promoted with this aim (36, 37). These traditional uses provide important clues and validation directions for modern pharmacological research on polysaccharides.

In conclusion, although various traditional uses from different parts have been extensively documented, the specific contributions of RNPs in these effects have not yet been systematically studied. The existing knowledge mostly reflects the synergistic effects of multiple active components. Therefore, future research should prioritize the use of separation-based bioassays and mechanisms of purified RNPs to clarify the specific role of RNPs in traditional applications.

## 3 Extraction and purification of RNPs

**Figure 2:**
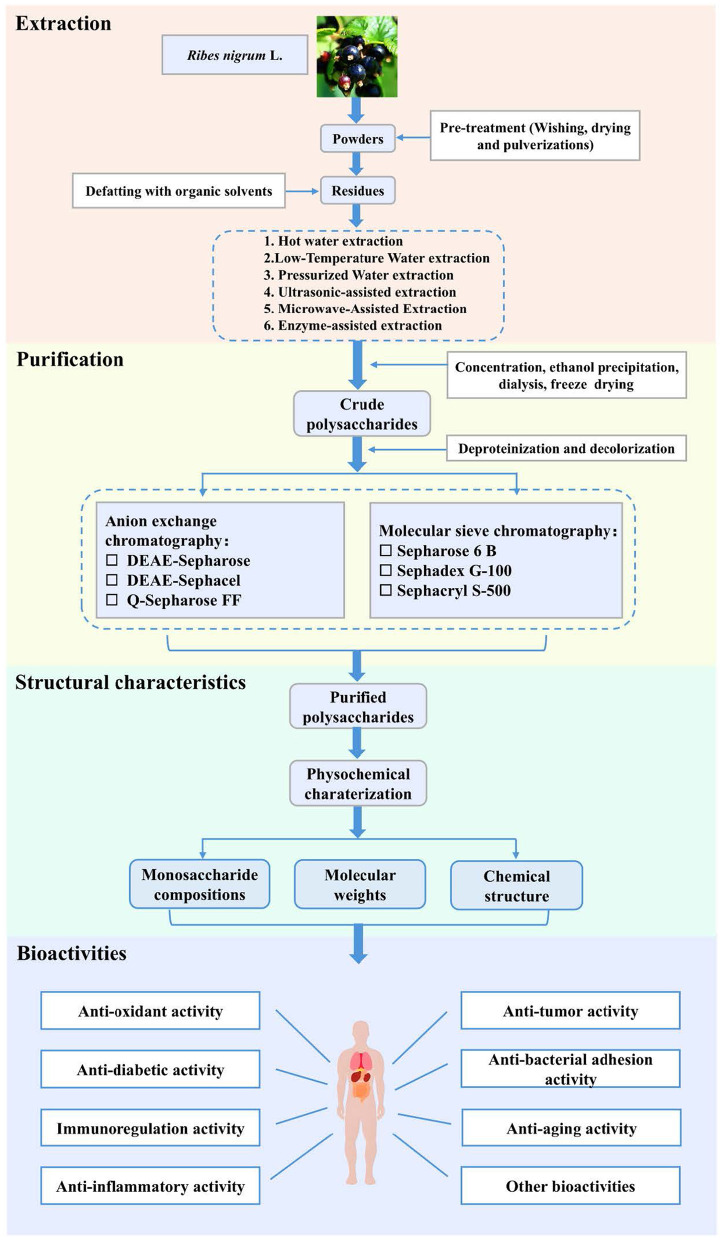
Schematic diagram of the extraction, purification, and biological activity of polysaccharides from *R. nigrum*.

Polysaccharides constitute one of the principal bioactive fractions in *R. nigrum*, consequently, their extraction and purification have attracted considerable research interest. Prior to extraction, the raw plant material is routinely subjected to a sequence of pretreatments—washing, drying, milling, sieving, and degreasing—aimed at eliminating colored contaminants, free monosaccharides, oligosaccharides, and other low-molecular weight metabolites (38, 39). Tables 1, 2 systematically summarized the extraction, purification, and structural characterization of RNPs, providing a comprehensive benchmark for current methods and their products. Table 1 highlights the diversity of extraction techniques and their optimization potential. Table 2 reveals the structural heterogeneity of purified polysaccharides, emphasizing how extraction conditions influence molecular weight, monosaccharide composition, and main-chain bonding patterns. Tailored extraction strategies hold promise for obtaining structurally defined polysaccharides with enhanced biological activity and functional specificity. Additionally, a detailed schematic diagram illustrating the extraction methods, purification, structural characteristics, and biological activities of RNPs is shown in Figure 2.

**Table 1:** Extraction and purification methods of polysaccharides from *R. nigrum*.

No	Extraction methods	Polysaccharides fraction	Source	Extraction condition	Extraction yield	Purification procedure	Impact on the structure	Refs.
1	Microwave-assisted extraction	PRNP-1	Fruit	Aqueous two-phase (ammonia sulfate 18.98 %, ethanol 26.75 %, w/w)	73.68 %	Sepharose 6 B column	Short-term rapid heating effectively reduces thermal degradation. The RG-I characteristic connection is retained, and the side chain integrity is good.	Huang et al. (58)
2		PRNP-2	Fruit	Aqueous two-phase (ammonia sulfate 18.98 %, ethanol 26.75 %, w/w)	77.75 %	Sepharose 6 B column		Huang et al. (58)
3		PRTP	Fruit	Dual-aqueous phase, 587 W, 60.5 °C, 9.5 min	13.08 %	Sepharose 6 B column		Yang et al. (59)
4		PRBP	Fruit	Dual-aqueous phase, 587 W, 60.5 °C, 9.5 min	42.65 %	Sepharose 6 B column		Yang et al. (59)
6		BCP, SBCP-1, SBCP-2, SBCP-3	Fruit	25 °C, 400 W, 25 min	ND	Q-Sepharose FF column, sulfation modification	Low power, slight heating, mild conditions are suitable for heat-sensitive polysaccharides, with minimal structural damage.	Xu et al. (54)
7		RNP	Fruit	3:1 (w/v), 60 °C, 80 W, 40 min	1.805 %	Ethanol precipitation		Luo et al. (120)
8		RNP	Fruit	1:20 (w/v), 400 W, 25 min	ND	Ethanol precipitation		Yu et al. (83)
9		BCP	Fruit	1:20 (w/v), 400 W, 25 min	ND	Q-Sepharose FF, Sephadex G-100 column		Xu et al. (63)
10		BCP-1	Fruit	1:20 (w/v), 400 W, 25 min	ND	Sephadex G-100 column		Xu et al. (55)
11		RNP	Fruit	1:29 (w/v), 57 °C, 100 W, 44 min	15.79 %	Ethanol precipitation		Huang et al. (121)
12	Ultrasound & enzyme assisted	PRCP, PRSP-1, PRSP-2, PRSP-3	Fruit	Enzymatic hydrolysis, ethanol precipitation	66.90 %	Sepharose 6 B column	The ultrasonic shear moderately degrades the main chain and exposes the active groups, while enzymatic hydrolysis specifically retains the RG-I side chain. The ratio of Rha/GalA is high, and the branch integrity is good.	Zhao et al. (53)
13		BCP-1	Fruit	40 °C, 45 min; enzymes (papain:pectinase=2:1, 40 °C, 45 min)	ND	Q-Sepharose FF, Sephadex G-100 column		Xu et al. (51)
14		RNP, CRNP-1, CRNP-2, CRNP-3	Fruit	600 W, 30 min; papain:pectinase=2:1	ND	Ethanol precipitation		Duan et al. (18)
15		BCP I	Fruit	26 min; papain:pectinase=2:1	14.28 %	Ethanol precipitation		Xu et al. (85)
16		BP, U-300, U-500, U-700	Fruit	Papain:pectinase=2:1, 50 °C, 50 min; ultrasound (300/500/700 W, 1 h)	ND	Sephacryl S-500 column		Yang et al. (50)
17	Enzyme-assisted extraction	CAPS	Fruit	Sclase S:Viscozyme=1:1, 48 °C, 6 h	0.004 %	Ethanol precipitation	Specificity determines the extent to which the structure is preserved.	Ashigai et al. (47)
18	Hot water extraction	BCP, DBCP-1, DBCP-2	Fruit	1:30 (w/v), 80 °C	ND	Ethanol precipitation	Shorter periods are preferable. Long-term high-temperature extraction leads to significant depolymerization and degradation of the main chain, and the side chains may be severely damaged.	Xu et al. (41)
19		RNP	Fruit	1:30 (w/v), 80 °C, 2 h, twice	4.32 %	Ethanol precipitation		Luo et al. (122)
20		BP, ABP-1, ABP-2, ABP-3	Fruit	1:4 (w/v), 1 h	7.12 %	Ethanol precipitation, acetylation modification		Xu et al. (43)
21		Pectin	Fruit	1:5 (w/v), 1 h	ND	Acetone precipitation		Konrade et al. (42)
22	Cold water extraction	RPS, F2	Seed	4 °C, three times	ND	DEAE-Sepharose column	The most gentle method retains the natural side chain structure and the terminal galactose epitope to the greatest extent, but has low efficiency.	Zippel et al. (71)
23		F2	Seed	8 °C, three times	0.3 %	DEAE-Sephacel column		Messing et al. (72)
24		RPS, WE, PBE	Seed	1:25 (w/v), 20 h, three times	1.4 %	DEAE-Sephace column		Lengsfeld et al. (96)
25	Pressurized water extraction	RNLP	Fruit	1.6 MPa, 52 °C, 51 min	11.68 %	Ethanol precipitation	The medium-pressure and low-temperature conditions are mild and can well preserve the polysaccharide structure. However, the pressure may cause some physical degradation.	Xu et al. (44)
26	Ion-exchange & precipitation	CAPS	Fruit	Amberlite IR-120B, IRA-410, SEP-PAK C-18 columns	ND	Ethanol precipitation	Without high temperature and strong shear, the structure is well preserved. However, it may affect the structure of the complex.	Takata et al. (64)

ND, not detected; PRNP-1, PRNP-2, PRTP, PRBP, PRCP, BCP, RNP, BCP-1, BCP I, BP, U-300, U-500, U-700, CAPS, DBCP-1, DBCP-2, RNLP, polysaccharide from *R. nigrum* fruits; PRSP-1, PRSP-2, PRSP-3, selenized polysaccharides from *R. nigrum* fruits; SBCP-1, SBCP-2, SBCP-3, sulfated polysaccharides from *R. nigrum* fruits; CRNP-1, CRNP-2, CRNP-2 carboxymethylated polysaccharides from *R. nigrum* fruits; ABP-1, ABP-2, ABP-3, acetylated polysaccharides from *R. nigrum* fruits; F2, RPS, WE, PBE, polysaccharides from *R. nigrum* seeds.

**Table 2:** Structural characteristics of the purified polysaccharide from *R. nigrum*.

Polysaccharides fraction	Extraction, isolation, and purification procedure	Source	Mw (kDa)	Monosaccharide composition (molar ratio)	Structural features	Structural characterization method	Reference
PRNP-1	Microwave-assisted aqueous two-phase (ammonia sulfate 18.98 % and ethanol 26.75 %, w/w) extraction, Sepharose 6 B column purification	Fruit	4.87 × 10^4^	Man, GalA, Glu, Gal, and Ara in a molar ratio of 14.74:12.38:7.35:45.59:19.94	( → 3)-α-L-Araf-(1 → 3)-β-D-Galp-(1 → 4)-β-D-Manp-(1 → 5)-α-Araf-(1 → 4)-α-D-GalpA-(1 → 3,6)-β-D-Glup-(1 →	HPLC, HPSEC, FT-IR, NMR, GC-MS, XRD, SEM, Methylation,	Huang et al. (58)
PRNP-2	Microwave-assisted aqueous two-phase (ammonia sulfate 18.98 % and ethanol 26.75 %, w/w) extraction, Sepharose 6 B column purification	Fruit	2.40 × 10^4^	Man, GalA, Glu, Gal, and Ara in a molar ratio of 7.43:20.46:6.33:34.36:34.43	( → 4)-β-D-Manp-(1 → 3)-α-D-Araf-(1 → 3)-β-D-Galp-(1 → 5)-α-Araf-(1 → 4)-α-D-GalpA-(1 → 3,6)-β-D-Glup-(1 →	HPLC, HPSEC, FT-IR, NMR, GC-MS, XRD, SEM, Methylation,	Huang et al. (58)
PRCP	Ultrasound-assisted enzymatic hydrolysis extraction, ethanol precipitation, Sepharose 6 B column purification	Fruit	2.04 × 10^4^	GalA, Rha, Ara, Man, Glc, and Gal in a molar ratio of 26.69:25.94:41.84:3.36:4.18:17.99	( → 2)α-L-Rhap-(1 → , α-L-Araf-(1 → , → 4)-α-D-GalAp-(1 → , → 4)-β-D-Manp-(1 → , → 6)-β-D-Galp-(1 → , and → 3,6)-β-D-Glcp-(1 →	FT-IR, NMR, SEM,	Zhao et al. (53)
PRSP-1	Ultrasound-assisted enzymatic hydrolysis extraction, ethanol precipitation, Sepharose 6 B column purification	Fruit	1.55 × 10^4^	GalA, Rha, Ara, Man, Glc, and Gal in a molar ratio of 26.67:21.33:33.33:7.67:9.00:22.00	→ 2)α-L-Rhap-(1 → , α-L-Araf-(1 → , → 4)-α-D-GalAp-(1 → , → 4)-β-D-Manp-(1 → , → 6)-β-D-Galp-(1 → , and → 3,6)-β-D-Glcp-(1 →	FT-IR, NMR, SEM,	Zhao et al. (53)
PRSP-2	Ultrasound-assisted enzymatic hydrolysis extraction, ethanol precipitation, Sepharose 6 B column purification	Fruit	1.29 × 10^4^	GalA, Rha, Ara, Man, Glc, and Gal in a molar ratio of 29.09:21.59:37.89:4.54:4.92:21.97	→ 2)α-L-Rhap-(1 → , α-L-Araf-(1 → , → 4)-α-D-GalAp-(1 → , → 4)-β-D-Manp-(1 → , → 6)-β-D-Galp-(1 → , and → 3,6)-β-D-Glcp-(1 →	FT-IR, NMR, SEM,	Zhao et al. (53)
PRSP-3	Ultrasound-assisted enzymatic hydrolysis extraction, ethanol precipitation, Sepharose 6 B column purification	Fruit	9.09 × 10^3^	GalA, Rha, Ara, Man, Glc, and Gal in a molar ratio of 30.86:22.47:37.45:4.49:4.12:20.61	( → 2)α-L-Rhap-(1 → , α-L-Araf-(1 → , → 4)-α-D-GalAp-(1 → , → 4)-β-D-Manp-(1 → , → 6)-β-D-Galp-(1 → , and → 3,6)-β-D-Glcp-(1 →	FT-IR, NMR, SEM,	Zhao et al. (53)
BCP-1	Ultrasound-assisted extraction (40 °C, 45 min), enzymes assisted extraction (papain:pectinase = 2:1, 40 °C, 45 min), ethanol precipitation, Q-Sepharose FF and Sephadex G-100 column purification	Fruit	14.05	GalA, Xyl, Man, Glu, and Gal in molar ratio of 1.00:3.14:1.83:17.90:1.98	ND	FT-IR, NMR, SEM,	Xu et al. (51)
CAPS	Enzymes assisted extraction (Sclase S: Viscozyme = 1:1, 48 °C, 6 h), ethanol precipitation	Fruit	27.643	ND	ND	ND	Ashigai et al. (47)
BCP	Hot water extraction (1:30 w/v, 80 °C), ethanol precipitation	Fruit	3.26 × 10^4^	Gal A, Rha, Ara, Man, Glu, and Gal in molar ratio of 0.66:0.32:1.00:0.19:0.09:0.31	ND	HPGPC, GC, FT-IR, NMR, SEM	Xu et al. (41)
DBCP-1	Hot water extraction (1:30 w/v, 80 °C), ethanol precipitation	Fruit	1.30 × 10^4^	Gal A, Rha, Ara, Man, Glu, and Gal in molar ratio of 2.49:0.71:1.00:0.41:0.11:0.63	ND	HPGPC, GC, FT-IR, NMR, SEM	Xu et al. (41)
DBCP-2	Hot water extraction (1:30 w/v, 80 °C), ethanol precipitation	Fruit	9.62 × 10^3^	Gal A, Rha, Ara, Man, Glu, and Gal in molar ratio of 1.27:0.67:1.00:0.86:0.56:0.97	ND	HPGPC, GC, FT-IR, NMR, SEM	Xu et al. (41)
BCP	ND	Fruit	3.26 × 10^4^	Gal A, Rha, Ara, Man, Glu, and Gal in molar ratio of 0.66:0.32:1.00:0.19:0.09:0.31	ND	HPLC, GC, FT-IR, NMR, SEM	Xu et al. (62)
U-400	ND	Fruit	1.89 × 10^4^	Gal A, Rha, Ara, Man, Glu, and Gal in molar ratio of 0.73:0.32:1.00:0.17:0.12:0.38	ND	HPLC, GC, FT-IR, NMR, SEM	Xu et al. (62)
U-600	ND	Fruit	1.32 × 10^4^	Gal A, Rha, Ara, Man, Glu, and Gal in molar ratio of 0.62:0.29:1.00:0.13:0.05:0.35	→ 5)-α-L-Araf-(1 → → )-α-L-Rhap-(1 → 4)-α-D-GalAp-(1 → 3, 6)-β-D-Glcp-(1 → 4)-α-D-GalAp-(1 → 5)-α-L-Araf-(1 →	HPLC, GC, FT-IR, NMR, SEM	Xu et al. (62)
CAPS	Enzymes assisted extraction (glucanase, 48 °C, 5 h), ethanol precipitation (three times)	Fruit	27.643	ND	ND	HPLC	Ashigai et al. (48)
BP	Enzymes assisted extraction (papain:pectinase=2:1, 50 °C, 50 min), ethanol precipitation	Fruit	8.13 × 10^3^	Rha, Ara, Man, Glu, and Gal in molar ratio of 27.24:46.91:3.81:4.18:17.86	α-L-Araf-(1 → , → 6)-β-D-Galp-(1 → , → 3,6)-β-D-Glcp-(1 → , → 4)-β-D-Manp-(1 → , → 2)-α-L-Rhap-(1 → , and → 4)-α-D-GalAp-(1 →	HPLC, GC, FT-IR, NMR	Yang et al. (50)
U-300	Enzymes assisted extraction (papain:pectinase = 2:1, 50 °C, 50 min), ethanol precipitation, ultrasound-assisted extraction (300 w, 1 h), Sephacryl S−500 column purification	Fruit	3.39 × 10^3^	Rha, Ara, Man, Glu, and Gal in molar ratio of 22.11:51.45:3.96:4.21:18.27	ND	HPLC, GC, FT-IR, NMR	Yang et al. (50)
U-500	Enzymes assisted extraction (papain:pectinase = 2:1, 50 °C, 50 min), ethanol precipitation, ultrasound-assisted extraction (500 w, 1 h), Sephacryl S−500 column purification	Fruit	1.74 × 10^3^	Rha, Ara, Man, Glu, and Gal in molar ratio of 27.77:43.73:5.59:7.39:15.52	ND	HPLC, GC, FT-IR, NMR	Yang et al. (50)
U-700	Enzymes assisted extraction (papain:pectinase = 2:1, 50 °C, 50 min), ethanol precipitation, ultrasound-assisted extraction (700 w, 1 h), Sephacryl S−500 column purification	Fruit	4.57 × 10^2^	Rha, Ara, Man, Glu, and Gal in molar ratio of 25.48:50.39:3.84:3.19:17.10	α-L-Araf-(1 → , → 6)-β-D-Galp-(1 → , → 3,6)-β-D-Glcp-(1 → , → 4)-β-D-Manp-(1 → , → 2)-α-L-Rhap-(1 → , and → 4)-α-D-GalAp-(1 →	HPLC, GC, FT-IR, NMR	Yang et al. (50)
RPS	Water extraction (4 °C, three times), ethanol precipitation	Seed	>1 × 10^3^	ND	ND	HPGPC, HPLC, GC-MS,	Zippel et al. (71)
F2	Water extraction (4 °C, three times), ethanol precipitation, DEAE Sepharose column purification	Seed	>1 × 10^3^	Ara, Gal, Xyl, Man, Glu, Rha, Fuc, Gal A, and Glu A in molar ratio of 42.9:24.4:20.1:4.3:3.6:2.6:1.4:0.4:0.3	ND	HPGPC, HPLC, GC-MS,	Zippel et al. (71)
PRTP	Microwave-assisted dual-aqueous phase extraction (1:58.5 w/v, 9.5 min, 60.5 °C, 587 W), ethanol precipitation, Sepharose 6 B column purification	Fruit	4.17 × 10^4^	Man, Rha, Gal A, Glu, Gal, and Ara in molar ratio of 13.54:6.00:11.18:6.15:44.39:18.74	→ 2,5)-α-L-Araf-(1 → 3)-α-D-Manp-(1 → 6)-β-D-Galp-(1 → 6)-α-D-Glcp-(1 → 4)-α-L-Rhap-(1 → 4)-α-D-GalAp-(1 →	HPLC, FT-IR, NMR, SEM	Yang et al. (59)
PRBP	Microwave-assisted dual-aqueous phase extraction (1:58.5 w/v,9.5 min, 60.5 °C, 587 W), ethanol precipitation, Sepharose 6 B column purification	Fruit	3.55 × 10^4^	Man, Rha, Gal A, Glu, Gal, and Ara in molar ratio of 6.36:5.30:19.40:5.27:33.30:30.37	( → 4)-α-L-Rhap-(1 → 3)-α-D-Manp-(1 → 6)-β-D-Galp-(1 → 6)-α-D-Glcp-(1 → 2,5)-α-L-Araf-(1 → 4)-α-D-GalAp-(1 →	HPLC, FT-IR, NMR, SEM	Yang et al. (59)
CAPS	Enzymes assisted extraction (Sclase S: Viscozyme = 1:1, 48 °C, 5 h), ethanol precipitation (three times)	Fruit	27.643	ND	ND	HPLC	Ashigai et al. (49)
BCP	Ultrasound-assisted extraction (25 °C, 400 W, 25 min), ethanol precipitation, Q-Sepharose FF column purification	Fruit	1.7656 × 10^4^	Rha, Ara, Xyl, Man, Glu, and Gal in molar ratio of 6.40:1.75:1.13:1.00:2.64:3.49	ND	HPGPC, GC, FT-IR, NMR	Xu et al. (54)
SBCP-1	Ultrasound-assisted extraction (25 °C, 400 W, 25 min), ethanol precipitation, Q-Sepharose FF column purification, sulfation modification	Fruit	1.8563 × 10^4^	Rha, Ara, Xyl, Man, Glu, and Gal in molar ratio of 1.78:3.44:0.59:1.00:1.17:2.51	ND	HPGPC, GC, FT-IR, NMR	Xu et al. (54)
SBCP-2	Ultrasound-assisted extraction (25 °C, 400 W, 25 min), ethanol precipitation, Q-Sepharose FF column purification, sulfation modification	Fruit	1.8264 × 10^4^	Rha, Ara, Xyl, Man, Glu, and Gal in molar ratio of 2.95:3.68:0.68:1.00:1.64:3.39	ND	HPGPC, GC, FT-IR, NMR	Xu et al. (54)
SBCP-3	Ultrasound-assisted extraction (25 °C, 400 W, 25 min), ethanol precipitation, Q-Sepharose FF column purification, sulfation modification	Fruit	1.7661 × 10^4^	Rha, Ara, Xyl, Man, Glu, and Gal in molar ratio of 4.02:2.16:2.09:1.00::1.12:1.42	ND	HPGPC, GC, FT-IR, NMR	Xu et al. (54)
RNP	Ultrasound-assisted extraction (600 W, 30 min), Enzymes assisted extraction (papain:pectinase = 2:1), ethanol precipitation,	Fruit	8.093 × 10^3^	Glu, Rha, Ara, Man, Gal, and Gal A in molar ratio of 1.00:2.31:13.29:0.95:5.13:1.96	ND	GC, HPLC, IR, SEM	Duan et al. (18)
CRNP-1	Ultrasound-assisted extraction (600 W, 30 min), Enzymes assisted extraction (papain:pectinase = 2:1), ethanol precipitation,	Fruit	1.2548 × 10^4^	Glu, Rha, Ara, Man, Gal, and Gal A in molar ratio of 1.00:2.09:8.29:1.34:3.50:0.96	ND	GC, HPLC, IR, SEM	Duan et al. (18)
CRNP-2	Ultrasound-assisted extraction (600 W, 30 min), Enzymes assisted extraction (papain:pectinase = 2:1), ethanol precipitation,	Fruit	1.1643 × 10^4^	Glu, Rha, Ara, Man, Gal, and Gal A in molar ratio of 1.00:1.55:4.5:1.09:5.24:1.28	ND	GC, HPLC, IR, SEM	Duan et al. (18)
CRNP-3	Ultrasound-assisted extraction (600 W, 30 min), Enzymes assisted extraction (papain:pectinase = 2:1), ethanol precipitation	Fruit	1.1036 × 10^4^	Glu, Rha, Ara, Man, Gal, and Gal A in molar ratio of 1.00:4.35:5.65:0.23:6.65:4.35	ND	GC, HPLC, IR, SEM	Duan et al. (18)
RNLP	Pressurized Water Extraction (1.6 MPa, 51 min, 52 °C), ethanol precipitation	Fruit	1.49 × 10^4^	Rha, Ara, Xyl, Man, Gal, and Glu in a molar ratio of 2.89:14.82:1.02:1.00:2.53:6.39	ND	GC, FT-IR	Xu et al. (44)
BCP I	Ultrasound-assisted extraction (26 min), Enzymes assisted extraction (papain:pectinase = 2:1), ethanol precipitation	Fruit	8.146 × 10^3^	Rha, Ara, Xyl, Man, Glu, and Gal in a molar ratio of 1.818:1.362:0.377:0.501:1.581:1.722	ND	HPLC, GC, FT-IR	Xu et al. (85)
CAPS	Amberlite IR-120B and IRA-410 columns remove ions, SEP-PAK C-18 column remove polyphenols, ethanol precipitation	Fruit	6 × 10^5^	Rha, Man, Ara, Gal, Xyl, and Glu in a molar ratio of 11.3:0.9:54.1:29.8:2.0:1.9	ND	HPLC	Takata et al. (64)
F2	Clod water extraction (8 °C, three times), ethanol precipitation, DEAE-Sephacel column purification	Seed	ND	Gal, Ara, Xyl, Man, Rha, Glu, and Fuc in a molar ratio of 28.3:24.5:15.7:3.5:2.4:1.0:0.6	ND	ND	Messing et al. (72)
pectin	Water extraction (1:5 w/v, 1 h,), acetone precipitation,	Fruit	115.75	Gal A, Ara, Gal, Man, Xyl, and Rha in a molar ratio of 55.5:16.7:10.5:7.4:6.8:3.1	ND	GPC, FT-IR-ATR,	Konrade et al. (42)
RPS	Water extraction (1:25 w/v, three times, 20 h), ethanol precipitation, DEAE-Sephace column purification	Seed	1072~35	Rha, Ara, Xyl, Man, Gal, and Glu in a molar ratio of 2:13:15:5:28:15	ND	PDA-HPLC, GC-MS	Lengsfeld et al. (96)
WE	Water extraction (1:25 w/v, three times, 20 h), ethanol precipitation, DEAE-Sephace column purification	Seed	1072~339	Rha, Ara, Xyl, Man, Gal, and Glu in a molar ratio of 1:10:2:5:24:46	ND	PDA-HPLC, GC-MS	Lengsfeld et al. (96)
PBE	Water extraction (1:25 w/v, three times, 20 h), ethanol precipitation, DEAE-Sephace column purification	Seed	107~26	Rha, Ara, Xyl, Man, Gal, and Glu in a molar ratio of 2:26:19:3:32:3	ND	PDA-HPLC, GC-MS	Lengsfeld et al. (96)
RNP	Hot water extraction (1:30 w/v, 80 °C, 2 h, twice), ethanol precipitation	Fruit	ND	ND	ND	ND	Luo et al. (122)
RNP	Ultrasound-assisted extraction (3:1 w/v, 60 °C, 80W, 40 min), ethanol precipitation	Fruit	ND	ND	ND	ND	Luo et al. (120)
RNP	Ultrasound-assisted extraction (1:20 w/v, 400 W, 25 min), ethanol precipitation	Fruit	ND	ND	ND	ND	Yu et al. (83)
BCP	Ultrasound-assisted extraction (1:20 w/v, 400 W, 25 min), ethanol precipitation, Q-Sepharose FF and Sephadex G-100 column purification	Fruit	16.329	GalA, Rha, Ara, Xyl, Man, Glu, and Gal in a molar ratio of 0.31:0.66:1.63: 0.36:0.23:0.31:1.00	ND	IR, GC	Xu et al. (63)
BCP-1	Ultrasound-assisted extraction (1:20 w/v, 400 W, 25 min), ethanol precipitation, Sephadex G-100 column purification	Fruit	51.88	Ara, Man, Glc, and Gal in a molar ratio of 9.08:1.00:15.77:0.41	ND	HPLC, GC, IR	Xu et al. (55)
RNP	Ultrasound-assisted extraction (1:29 w/v, 57 °C, 100 W, 44 min), ethanol precipitation	Fruit	ND	ND	ND	ND	Huang et al. (121)
BP	Water extraction (1:4 w/v, 1 h), ethanol precipitation	Fruit	ND	GalA, Rha, Ara, Xyl, Man, Glc, and Gal in molar ratio of 4.85:17.19:21.68:3.74:0.57:10.40:41.57	ND	GC, FT-IR, SEM	Xu et al. (43)
ABP-1	Water extraction (1:4 w/v, 1 h), ethanol precipitation, acetylation modification	Fruit	ND	GalA, Rha, Ara, Xyl, Man, Glc, and Gal in molar ratio of 8.88:25.57:30.17:5.01:5.46:8.59:16.30	ND	GC, FT-IR, SEM	Xu et al. (43)
ABP-2	Water extraction (1:4 w/v, 1 h), ethanol precipitation, acetylation modification	Fruit	ND	GalA, Rha, Ara, Xyl, Man, Glc, and Gal in molar ratio of 10.23:16.76:26.86:8.56:5.11:11.88:20.61	ND	GC, FT-IR, SEM	Xu et al. (43)
ABP-3	Water extraction (1:4 w/v, 1 h), ethanol precipitation, acetylation modification	Fruit	ND	GalA, Rha, Ara, Xyl, Man, Glc, and Gal in molar ratio of 14.99:15.50:31.64:8.23:3.46:7.01:19.18	ND	GC, FT-IR, SEM	Xu et al. (43)
BCP	Deep fusion solvent extraction; 80 % ethanol precipitation; HP-20 macroporous resin and Sephadex G-100 gel purification.	Fruit	240.508	GalA, Rha, Ara, GlcA, Glc, Gal, and Man in a molar ratio of 44.15:1.76:14.50:3.00:14.40:17.10:5.09	39.28	GPC, PMP-HPLC, SEM	Zhu et al. (86)

ND, not detected; PRNP-1, PRNP-2, PRCP, BCP-1, CAPS, BCP, DBCP-1, DBCP-2, BCP, U-400, U-600, CAPS, BP, U-300, U-500, U-700, PRTP, PRBP, RNP, RNLP, BCP I, polysaccharide from *R. nigrum* fruits; PRSP-1, PRSP-2, PRSP-3, selenized polysaccharides from *R. nigrum* fruits; RPS, F2, WE, PBE, polysaccharides from *R. nigrum* seeds; SBCP-1, SBCP-2, SBCP-3, sulfated polysaccharides from *R. nigrum* fruits; CRNP-1, CRNP-2, CRNP-3, carboxymethylated polysaccharides from *R. nigrum* fruits; ABP-1, ABP-2, ABP-3, acetylated polysaccharides from *R. nigrum* fruits.

### 3.1 Extraction of RNPs

A variety of extraction techniques have been employed for the recovery of RNPs, including hot-water extraction (HWE), enzyme-assisted extraction (EAE), ultrasound-assisted extraction (UAE), microwave-assisted extraction (MAE), and others. The extraction efficiency of RNPs is significantly dependent on the method applied. Each approach presents distinct advantages and limitations; therefore, systematic evaluation of multi-method synergistic strategies is warranted to further optimize RNP yield.

#### 3.1.1 Hot water extraction (HWE)

As a conventional and widely used technique, HWE is frequently employed for the isolation of polysaccharides from natural sources due to its operational simplicity, cost-effectiveness, and broad applicability (40). However, this method is often associated with prolonged extraction time and relatively low efficiency. In the case of *R. nigrum*, several polysaccharide fractions have been successfully extracted using HWE under optimized conditions. For instance, black currant polysaccharide (BCP), DBCP-1, and DBCP-2 were extracted from fruit bodies using hot water (1:30, w/v) at 80°C, followed by ethanol precipitation (41). Similarly, pectin and BP were obtained by water extraction (1:5–1:4, w/v) at elevated temperatures, and further purified via acetone or ethanol precipitation (42, 43). Another study reported the extraction of RNLP using pressurized hot water (1.6 MPa, 52°C, 51 min), demonstrating the adaptability of water-based methods under moderate pressure (44). These examples confirm that HWE remains a fundamental and frequently applied technique for polysaccharide extraction from *R. nigrum*, particularly when combined with subsequent purification steps such as column chromatography or solvent precipitation.

#### 3.1.2 Enzyme-assisted extraction (EAE)

As a green and efficient extraction strategy, enzyme-assisted extraction (EAE) has gained increasing attention for its ability to enhance polysaccharide yield under mild conditions, while preserving structural integrity. This method typically involves the use of specific enzymes such as cellulase, pectinase, papain, or complex enzyme systems to degrade cell wall components, thereby facilitating the release of polysaccharides (45, 46). In the case of *R. nigrum*, several polysaccharide fractions have been isolated using EAE under optimized conditions. For example, the fraction CAPS was extracted using a combined enzyme system (Sclase S:Viscozyme = 1:1) at 48°C for 5~6 h, followed by ethanol precipitation (47–49). Similarly, BP, U-300, U-500, and U-700 were obtained by enzymatic treatment with papain and pectinase (2:1, w/w) at 50°C for 50 min, followed by ethanol precipitation, with some fractions further purified by ultrasound-assisted extraction and Sephacryl S-500 column chromatography (50). Another study reported the extraction of BCP-1 using a dual enzyme system (papain:pectinase = 2:1) at 40°C for 45 min, combined with ultrasonication and column purification (51). These examples demonstrate that EAE is a versatile and effective method for extracting polysaccharides from *R. nigrum*, often yielding fractions with well-defined structural characteristics and bioactivities.

#### 3.1.3 Ultrasonic-assisted extraction (UAE)

Ultrasound-assisted extraction (UAE) is recognized as a green and high-efficiency strategy for polysaccharide recovery; acoustic cavitation disrupts plant cell walls, shortens processing time and increases yield (52). *R. nigrum* have been extensively used to validate the technique. Fruit-body material supplied PRCP, PRSP-1, PRSP-2 and PRSP-3 after UAE-assisted enzymatic hydrolysis, ethanol precipitation and Sepharose 6 B chromatography (53). Under mild conditions (25°C, 400 W, 25 min) the same approach afforded neutral β-glucan BCP and its sulphated derivatives SBCP-1, SBCP-2 and SBCP-3 following ion-exchange clean-up (54). Comparable protocols (600 W, 30 min) yielded heteropolysaccharides RNP and the CRNP series after enzymatic digestion and alcoholic precipitation (18), whereas a 400 W, 25 min treatment released BCP-1 that was subsequently purified on Sephadex G-100 (55). Collectively, these studies demonstrate that UAE reliably delivers Morchella polysaccharides with well-defined molecular weights and structural fingerprints suited to detailed bioactivity evaluation.

#### 3.1.4 Microwave-assisted extraction (MAE)

As an advanced and energy-efficient extraction technique, microwave-assisted extraction (MAE) has gained prominence in the field of polysaccharide isolation for its rapid heating capacity and improved extraction efficiency (56). The method utilizes microwave energy to disrupt plant and fungal cell walls, thereby promoting the release of bioactive compounds (57). In the case of *R. nigrum*, several polysaccharide fractions have been effectively extracted using MAE under optimized parameters. For example, PRNP-1 and PRNP-2 were obtained through microwave-assisted aqueous two-phase extraction (MA-ATPE) with ammonia sulfate (18.98 %) and ethanol (26.75 %, w/w), followed by purification on a Sepharose 6 B column (58). Similarly, PRTP and PRBP were isolated using microwave-assisted dual-aqueous phase extraction under conditions of 587 W, 60.5°C, and 9.5 min, with subsequent ethanol precipitation and column purification (59). Notably, MA-ATPE not only achieves clear phase separation but also significantly enhances extraction efficiency. A quantitative comparison of different extraction methods for blackcurrant polysaccharides revealed that MA-ATPE gave the highest total polysaccharide yield (55.73 ± 1.35 %) after only 10 min of extraction, whereas conventional aqueous two-phase extraction (ATPE), UAE, and MAE produced yields of 28.56 %, 40.12 %, and 43.24 %, respectively. This represents an approximately 95 % increase over conventional ATPE and a 39 % increase over UAE, highlighting the unique advantage of combining microwave irradiation with an ATPE (59). These studies demonstrate that MAE not only shortens extraction time and increase the yield, but also yields polysaccharides with specific structural features, such as well-defined glycosidic linkages and monosaccharide compositions. Therefore, MAE represents a highly efficient and controllable method for the extraction of polysaccharides from *R. nigrum*, particularly when combined with appropriate phase systems and purification steps.

#### 3.1.5 Different extraction methods on structural integrity

For pectin-type polysaccharides, their biological activity largely depends on the branching structure, particularly the integrity of the rhamnogalacturonan I (RG-I) domain. The abundance of GalA and Rha in RNPs indicates the presence of RG-I regions. Therefore, when comparing different extraction methods, attention should not be confined solely to extraction yield; instead, it is essential to critically evaluate the impact of each extraction process on the integrity of side chains and the structure of the backbone (60, 61).

Although HWE is considered mild, prolonged exposure to high temperatures may promote β-elimination reactions, leading to degradation of homogalacturonan regions and release of neutral side chains (41, 42) The natural pH of blackcurrant fruit is approximately 3~4, and heating may catalyze the hydrolysis of acid-labile arabinofuranosyl linkages, resulting in the loss of arabinan-rich side chains. For instance, the BCP obtained by hot water extraction at 80°C for 2 h exhibited a GalA/Rha molar ratio of 0.66:0.32 (approximately 0.48). In contrast, some fractions obtained by enzyme-assisted extraction showed higher ratios, suggesting that hot water extraction compromises the integrity of RG-I domains (41, 53). UAE induces mechanical shear forces via sonication, which can cleave glycosidic bonds. Low-intensity ultrasound preserves RG-I characteristics, whereas high-intensity ultrasound yields lower molecular weight fractions with altered monosaccharide profiles (18, 50). An increase in reducing sugar content directly indicates chain scission (62). Medium-intensity ultrasound achieves a favorable balance between extraction efficiency and structural integrity, yielding BCP that retains detectable RG-I linkages (54, 63). In EAE, the specificity of the enzyme system determines the extent of structural preservation. Pectinase cleaves α-1,4 glycosidic bonds in HG regions, releasing RG-I-enriched fragments, thereby potentially enriching branched structures. Cellulase acts solely on cellulose without disrupting RG-I side chains and is currently the most structure-preserving option. Papain is primarily used for deproteinization and does not act on glycosidic bonds, thus well preserving the native structure. In contrast, mixed enzyme systems (e.g., Sclase S:Viscozyme = 1:1), despite high extraction efficiency, contain multiple glycosidases that can cause significant debranching. For example, the mixed enzyme-extracted CAPS exhibited lower molecular weight but retained the active epitopes required for TLR4 recognition (47, 64). MAE enables rapid volumetric heating, which accelerates extraction, but localized overheating may degrade thermolabile side chains. Under optimized conditions, MAE-derived PRTP and PRBP retained RG-I features, and NMR confirmed the presence of → 2)-α-L-Rhap-(1 → 4)-α-D-GalpA-(1 → linkages (58, 59). Short extraction times effectively reduce thermal degradation. In summary, the choice of extraction method necessitates a trade-off between extraction yield and structural integrity.

### 3.2 Purification of RBPs

#### 3.2.1 Deproteinization

Deproteinization is commonly achieved by enzymatic hydrolysis, the Sevag protocol, trichloroacetic-acid (TCA) precipitation, trifluorotrichloroethane (TTE) extraction or macroporous-resin adsorption (65). Enzymatic removal is highly efficient and minimizes polysaccharide loss, but the cost is prohibitive for large-scale use (66). The Sevag method (chloroform-n-butanol) is rapid, inexpensive and gentle, yet repeated extractions are required and efficacy drops when protein-polysaccharide complexes are abundant (67). TCA precipitates proteins quantitatively while preserving the carbohydrate backbone, but the acidic environment may partially hydrolyse acid-labile polysaccharides (68). TTE affords single-step deproteinization without impairing biological activity; however, its volatility and hepatotoxicity demand stringent personal protection and hazardous-waste management. Macroporous resins combine operational simplicity, low solvent consumption and high adsorption selectivity to deliver proteins-free polysaccharides in a single pass (69). Because no protocol is universally superior, the choice must balance sample complexity, downstream applications, economic constraints and safety regulations to maximize efficiency while safeguarding product integrity.

#### 3.2.2 Chromatographic column separation

Chromatographic column-based separation and purification is one of the most widely used techniques for obtaining homogeneous polysaccharide fractions, primarily involving ion-exchange chromatography and size-exclusion chromatography (70). Ion-exchange chromatography separates molecules based on their charge properties and is commonly applied in the purification of biological macromolecules such as polysaccharides, proteins, and nucleic acids. In the purification of RNPs, columns such as Q-Sepharose FF, DEAE-Sepharose, and DEAE-Sephacel have been frequently employed. For instance, BCP-1 was purified using Q-Sepharose FF and Sephadex G-100 columns (51), while F2 and related fractions from seeds were isolated using DEAE-Sepharose or DEAE-Sephacel columns (71, 72). Size-exclusion chromatography, also known as gel filtration, separates molecules according to their hydrodynamic volume and molecular weight. Commonly used media for polysaccharide purification include Sepharose 6 B, Sephadex G-100, Sephacryl S-500, and Sepharose CL. Examples include PRNP-1 and PRNP-2, purified using Sepharose 6 B (58), and U-300, U-500, and U-700, further purified on a Sephacryl S−500 column (50). In practice, a combination of ion-exchange and size-exclusion chromatography is often employed to achieve high-purity polysaccharide fractions. For example, BCP was sequentially purified using Q-Sepharose FF and Sephadex G-100 columns (63), demonstrating that the integrated approach effectively yields polysaccharides with well-defined structural characteristics and high purity suitable for subsequent structural and functional analyses.

## 4 Structural characterization

**Figure 3:**
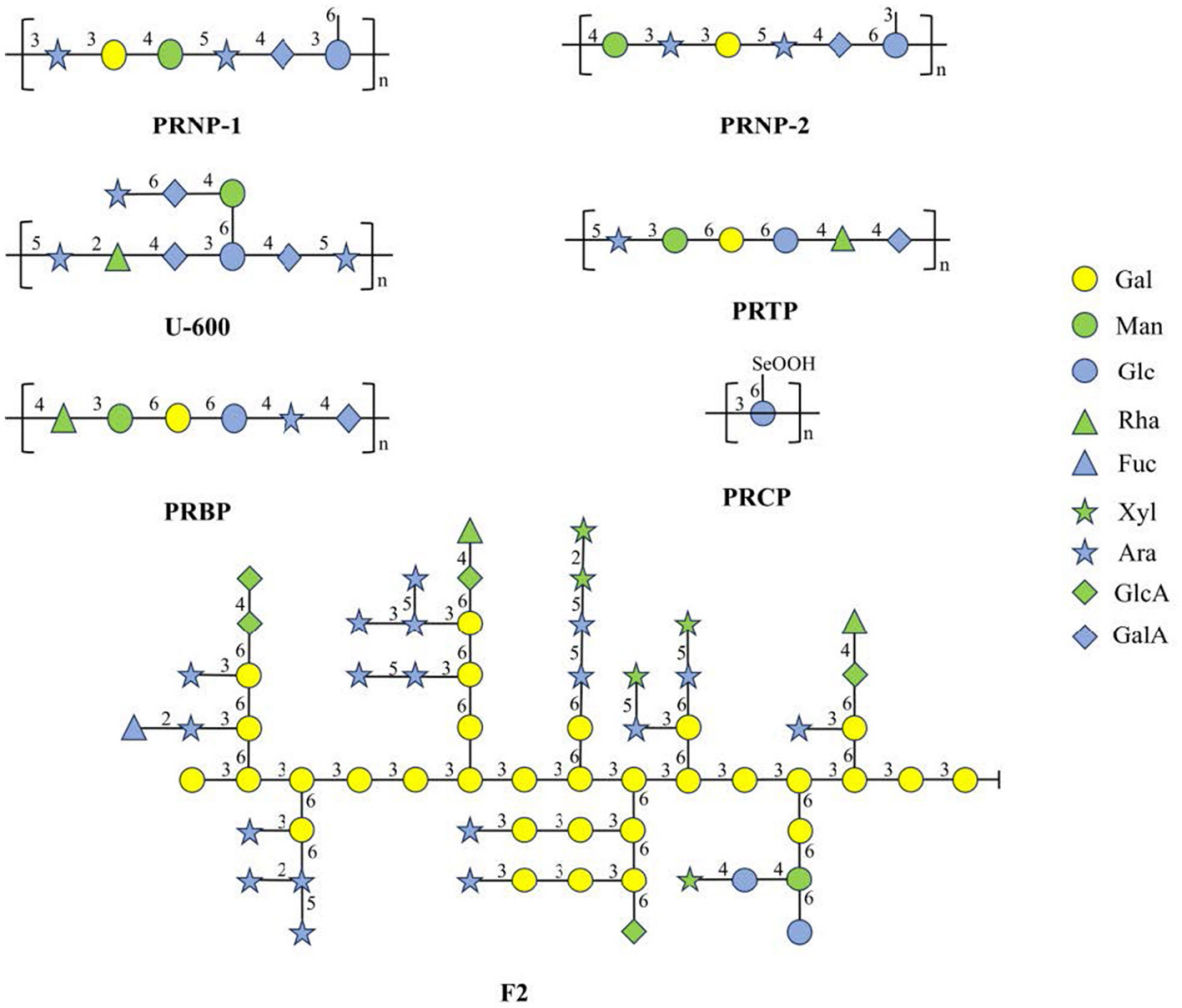
Schematic representation of reported RNP structural fragments (53, 58, 59, 62, 72).

Elucidating the precise structural framework of polysaccharides is essential for understanding their physicochemical properties and biological functions. Figure 3 summarizes the representative structural fragments of RNPs reported in the literature in a schematic form. The labels (PRNP-1, PRNP-2, U-600, PRTP, PRBP, F2) respectively represent the fragment types or structural features identified in different literature. The key structural information of polysaccharides is not only critical for establishing structure-activity relationships but also provides essential guidance for subsequent modification and application in the fields of biomedicine and materials science.

### 4.1 Molecular weight

Molecular weight is a fundamental physicochemical property of polysaccharides, commonly determined by techniques such as high-performance liquid chromatography (HPLC) and high-performance gel permeation chromatography (HPGPC) (73). Among polysaccharides derived from *R. nigrum*, molecular weights exhibit considerable variation, reflecting differences in extraction methods and source materials. For instance, the molecular weights of purified fractions range from as low as 4.57 × 10^2^ kDa for U-700 to 6.00 × 10^5^ kDa for CAPS. Specific examples include PRNP-1 and PRNP-2, which have molecular weights of 4.87 × 10^4^ kDa and 2.40 × 10^4^ kDa, respectively, as determined by HPSEC and HPLC (58). Ultrasound-assisted extraction typically yielded fractions with moderate molecular weights, such as PRSP-3 at 9.09 × 10^3^ kDa (53) and BCP at 3.26 × 10^4^ kDa (41). Regarding enzymatically extracted polysaccharides, they showed variable molecular weights, including BP at 8.13 × 10^3^ kDa and U-300 at 3.39 × 10^3^ kDa (50). It is noteworthy that even polysaccharides derived from the same species and extraction method can exhibit significant differences in molecular weight due to variations in purification procedures. Taking the hot water-extracted fractions as an example, the molecular weights of BCP, DBCP-1, and DBCP-2 were 3.26 × 104 kDa, 1.30 × 10^4^ kDa, and 9.62 × 10^3^ kDa, respectively (41). These observations highlight that both the extraction strategy and subsequent purification steps critically influence the molecular weight of the final polysaccharide product, thereby affecting its structural and potential functional properties.

### 4.2 Monosaccharide composition

Monosaccharide composition serves as a critical structural feature of polysaccharides, typically analyzed through techniques such as gas chromatography (GC), HPLC, gas chromatography-mass spectrometry (GC-MS), and high-performance anion-exchange chromatography (HPAEC) (74). In polysaccharides derived from *R. nigrum*, the predominant monosaccharides include rhamnose (Rha), arabinose (Ara), glucose (Glc), galactose (Gal), mannose (Man), xylose (Xyl), and galacturonic acid (GalA), with molar ratios varying considerably across different fractions. For example, PRNP-1 was found to contain Man, GalA, Glu, Gal, and Ara in a molar ratio of 14.74:12.38:7.35:45.59:19.94, as determined by GC-MS (58). Another fraction, PRCP, extracted using ultrasound-assisted enzymatic hydrolysis, consisted of GalA, Rha, Ara, Man, Glc, and Gal in a molar ratio of 26.69:25.94:41.84:3.36:4.18:17.99 (53). Polysaccharides obtained via hot water extraction, such as BCP, exhibited a distinct monosaccharide profile, with GalA, Rha, Ara, Man, Glu, and Gal in a molar ratio of 0.66:0.32:1.00:0.19:0.09:0.31 (41). Enzymatically extracted fractions also displayed notable compositional diversity: BP contained Rha, Ara, Man, Glu, and Gal in a ratio of 27.24:46.91:3.81:4.18:17.86 (50), while CAPS isolated using ion-exchange chromatography showed Rha, Man, Ara, Gal, Xyl, and Glu in a molar ratio of 11.3:0.9:54.1:29.8:2.0:1.9 (64).

Across all the reported RNP fractions, three regularities can be summarized. First, Rha, Ara, and Gal are universally present in every RNP fraction described to date, suggesting that these three monosaccharides constitute the invariant core of RNPs. Second, GalA, while common, is not an absolute constituent: it is absent in BP (50) and CAPS (64), indicating that some RNPs are neutral polysaccharides without uronic acid residues. Third, Xyl occurs only sporadically, and the molar ratios of mannose and glucose vary widely, implying that these sugars are accessory components that may be introduced or enriched by specific extraction procedures. These compositional differences highlight the influence of extraction and purification methods on monosaccharide makeup, which may further affect the physicochemical properties and biological activities of RNPs.

### 4.3 Structural features

Structural characterization is essential for understanding the bioactivity of polysaccharides, with analytical techniques including Fourier-transform infrared spectroscopy (FT-IR), nuclear magnetic resonance (NMR), GC-MS, scanning electron microscopy (SEM), and HPLC being widely employed (75). Among the purified polysaccharides from *R. nigrum* species, detailed structural features have been reported for several fractions. For example, PRNP-1 was identified to possess a backbone composed of → 3)-α-L-Araf-(1 → 3)-β-D-Galp-(1 → 4)-β-D-Manp-(1 → 5)-α-Araf-(1 → 4)-α-D-GalpA-(1 → 3,6)-β-D-Glup-(1 → , as determined through methylation analysis and NMR spectroscopy (58). Similarly, PRTP obtained via microwave-assisted dual-aqueous phase extraction exhibited a structure characterized by → 2,5)-α-L-Araf-(1 → 3)-α-D-Manp-(1 → 6)-β-D-Galp-(1 → 6)-α-D-Glcp-(1 → 4)-α-L-Rhap-(1 → 4)-α-D-GalAp-(1 → (59). In enzymatically extracted polysaccharides such as BP, multiple linkage types were identified, including α-L-Araf-(1 → , → 6)-β-D-Galp-(1 → , → 3,6)-β-D-Glcp-(1 → , → 4)-β-D-Manp-(1 → , → 2)-α-L-Rhap-(1 → , and → 4)-α-D-GalAp-(1 → (50). The fraction U-600 was also reported to feature a backbone of → 5)-α-L-Araf-(1 → → )-α-L-Rhap-(1 → 4)-α-D-GalAp-(1 → 3,6)-β-D-Glcp-(1 → 4)-α-D-GalAp-(1 → 5)-α-L-Araf-(1 → (62).

These structural features collectively determine the typical physical and chemical properties of RNPs. It is worth noting that the arrangement pattern of glycosidic bonds also has an inseparable influence on biological activity. For example, the alternating sequence in the main chain of PRNP-2 → 4)-β-D-Manp-(1 → 3)-α-D-Araf-(1 → and → 4)-α-D-GalpA-(1 → 3,6)-β-D-Glup-(1 → generates a mixed flexible-rigid structural conformation. This enables the Araf-GalpA fragment to deeply insert into the catalytic channel of xanthine oxidase (XOD), promoting the transformation of the α-helix to a β-sheet, thereby effectively inhibiting XOD to exert anti-gout activity (58). Similarly, we observed these structural features in RNPs, including molecular weight, monosaccharide composition, and glycosidic bond types, which are crucial for understanding the structure-activity relationship of RNPs.

### 4.4 Conformational features

Conformational characteristics of polysaccharides play a critical role in determining their biological functions. Techniques such as SEM, X-ray diffraction (XRD), atomic force microscopy (AFM), and Congo red staining are commonly employed to investigate polysaccharide conformation and morphology (76).

Several polysaccharides from *R. nigrum* species have been characterized using these approaches. SEM analyses revealed distinct surface morphologies among different fractions. For instance, PRNP-1 exhibited a flaky and rough surface with irregular grids and pores, while PRNP-2 showed a smooth and flaky morphology (58). PRCP displayed a dense and rough flaky structure, whereas the selenized derivatives (PRSPs) presented loose sheets with smoother surfaces and smaller fragments, indicating that selenization significantly influenced polysaccharide morphology (53). Similarly, BCP-1 was observed to form aggregates with a honeycomb-like surface (51), and both BCP and DBCP exhibited flaky structures with smooth surfaces, though degraded fractions showed reduced connectivity due to oxidative treatment (41). In the case of microwave-extracted fractions, PRTP and PRBP both showed smooth and compact sheet structures, with PRTP possessing a larger and more integrated surface (59). Ultrasonication was also found to cause fragmentation of original flakes into smaller pieces, attributed to cavitation and shear forces (62).

The triple-helix conformation, a structure associated with immunomodulatory and antioxidant activities, can be identified through Congo red binding experiments. Although not all RNPs have been fully characterized in this regard, the combination of SEM with techniques such as FT-IR and NMR has enabled preliminary understanding of their chain arrangement and higher-order structure. For example, methylation and GC-MS analyses indicated that PRNP-1 contains a complex backbone with multiple glycosidic linkages (58), while structural studies on U-600 revealed a defined repeating unit (62).

These observations highlight that conformational features—such as chain flexibility, surface topology, and helical organization—are closely influenced by extraction methods and purification procedures. Chemical modifications, including selenylation, sulfation, carboxymethylation, and acetylation, further alter polysaccharide morphology and conformational behavior, as evidenced by changes in surface roughness, porosity, and molecular packing. Importantly, these conformational and morphological alterations serve as the intermediary link through which structural modifications translate into enhanced or diversified biological activities (e.g., antioxidant, immunomodulatory, and enzyme inhibitory effects), as will be detailed in the following section. Further systematic studies using advanced microscopy and spectroscopic tools will be essential to establish clear structure-function relationships for RNPs and to facilitate their potential application in functional foods and pharmaceuticals.

### 4.5 Structural modifications

Structural modification of polysaccharides serves as a strategic approach to enhance their physicochemical properties and biological activities, primarily through physical, chemical, and biological methods. Among RNPs, several modification strategies have been successfully implemented to improve their functional characteristics (77, 78).

Selenation is one of the most extensively studied chemical modifications, capable of significantly enhancing the biological activity of polysaccharides (79). The PRSP series were synthesized from PRCP using the nitric acid-sodium selenite method under controlled ultrasonic conditions. This process led to significant alterations in surface morphology and reduced molecular weight due to acidic degradation (53). In another study, RP was effectively functionalized with selenium nanoparticles (SeNPs) through Se-O bonds, as confirmed by FT-IR spectroscopy showing a distinct shift in the O-H absorption peak, indicating successful interaction between the polysaccharide and SeNPs (80).

Sulfation and carboxymethylation have also been successfully applied to introduce functional groups. The SBCP-1 to SBCP-3 fractions were prepared through sulfation modification, producing polysaccharide derivatives with potentially enhanced bioactivities (54). Carboxymethylation of RNP was performed using isopropanol, sodium hydroxide, and monochloroacetic acid, yielding CRNP derivatives. Although GC analysis indicated that both native and carboxymethylated RNPs were heteropolysaccharides lacking a triple-helix conformation, SEM imaging revealed that the flaky structures with uneven surfaces were preserved, suggesting the modification did not substantially alter the overall morphology (18).

Acetylation modification of BP resulted in notable morphological changes. While native BP displayed a smooth, flaky structure, its acetylated derivatives (ABP-1 to ABP-3) exhibited irregular shapes, increased surface roughness, and porous structures, with the extent of modification dependent on the substitution degree (43).

These modifications demonstrate that targeted structural alterations—including selenization, sulfation, carboxymethylation, and acetylation—can effectively modulate the physicochemical and conformational properties of RNPs. Such modifications often correlate with improved functional performance, including enhanced antioxidant capacity, bioavailability, and targeting potential. However, current research has primarily focused on single modification types, while the relationships between complex modifications and *in vivo* bioactivities still require systematic exploration. Further research is essential to elucidate precise structure-activity relationships and expand the applications of modified RNPs in functional foods and pharmaceutical formulations.

## 5 Pharmacological properties

**Figure 4:**
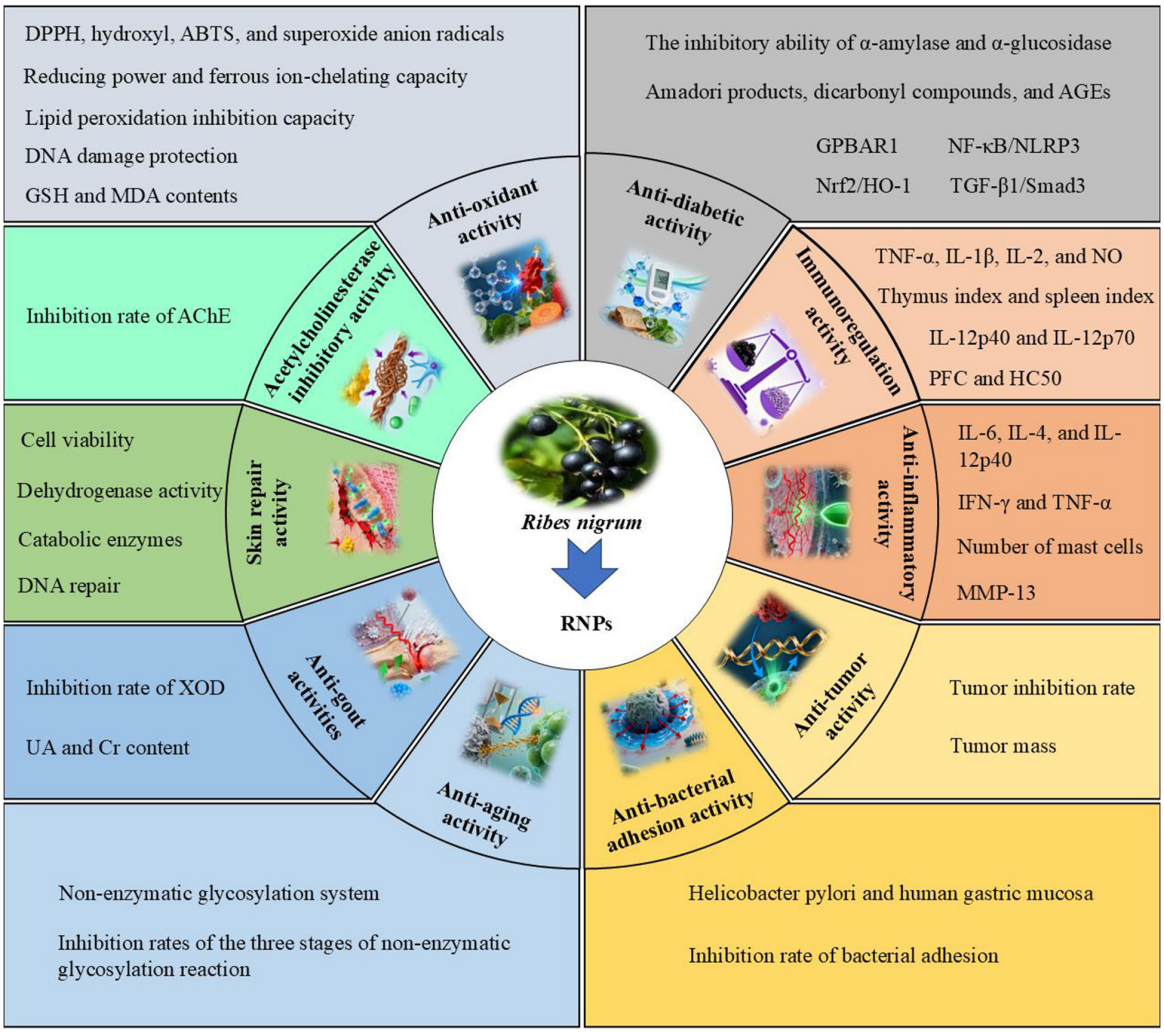
An overreview of biological activities and the possible molecular mechanism of RNPs.

As an important natural active substance extracted from *R. nigrum*, RNPs have continued to rise in the research of food science and medical care in recent years. A large number of modern pharmacological experiments and preclinical studies have confirmed that RNPs are not a single functional active ingredient, but exhibit a very broad and significant biological activity spectrum. These activities include anti-oxidant activity, anti-diabetic activity, immunoregulation activities, anti-inflammatory activities, anti-tumor activity, anti-bacterial adhesion activity, anti-aging activity, anti-gout activities, skin repair activity, and acetylcholinesterase inhibitory activity. It is worth further studying its mechanism of action and potential value. In this study, we will explore these pharmacological effects and systematically collate the biological activity information of different RNPs in Table 3. At the same time, we elucidated all the pharmacological properties and potential molecular mechanisms of RNPs in Figure 4.

**Table 3:** Biological activities of polysaccharides purified from *R. nigrum Polysaccharides*. (Increase, ↑; Decrease, ↓).

Biological activities	Name	Types	Testing subjects	Doses/Duration	Effects/Mechanisms	References
Anti-oxidant activity						
	DBCP-1	*In vitro*	DPPH, hydroxyl, and superoxide anion radicals, reducing power, ferrous ion-chelating capacity	0.2, 0.4, 0.6, 0.8, 1.0, and 1.2 mg/mL	The IC_50_ values of DPPH, hydroxyl free, and superoxide anion radical were 0.54, 0.33, and 0.76 mg/mL, respectively. The reduction ability is 0.252. The chelation ability was 22.28 ± 2.12.	Xu et al. (41)
	DBCP-2	*In vitro*	DPPH, hydroxyl, and superoxide anion radicals, reducing power, ferrous ion-chelating capacity	0.2, 0.4, 0.6, 0.8, 1.0, and 1.2 mg/mL	The IC_50_ values of DPPH, hydroxyl free, and superoxide anion radical were 0.40, 0.27, and 0.57 mg/mL, respectively. The reduction ability is 0.414. The chelation ability was 23.32 ± 1.30.	Xu et al. (41)
	BCP	*In vitro*	Hydroxyl and superoxide anion radicals, Lipid peroxidation inhibition capacity, DNA damage protection	0.2, 0.4, 0.6, 0.8, 1.0, and 1.2 mg/mL 0.5, 1.0, 1.5, 2.0, 3.0, and 4.0 mg/mL	It has scavenging ability to hydroxyl radical and superoxide radical. Lipid peroxidation inhibition rate was 35.34 ± 1.02 %. The DNA protection rate was 30.32 ± 0.93 %.	Xu et al. (62)
	U-400	*In vitro*	Hydroxyl and superoxide anion radicals, Lipid peroxidation inhibition capacity, DNA damage protection	0.2, 0.4, 0.6, 0.8, 1.0, and 1.2 mg/mL 0.5, 1.0, 1.5, 2.0, 3.0, and 4.0 mg/mL	The IC_50_ values for hydroxyl radical and superoxide radical were 0.31 and 0.95 mg/mL, respectively. Lipid peroxidation inhibition rate was 44.38 ± 0.77 %. The DNA protection rate was 42.66 ± 1.02 %.	Xu et al. (62)
	U-600	*In vitro*	Hydroxyl and superoxide anion radicals, Lipid peroxidation inhibition capacity, DNA damage protection	0.2, 0.4, 0.6, 0.8, 1.0, and 1.2 mg/mL 0.5, 1.0, 1.5, 2.0, 3.0, and 4.0 mg/mL	The IC_50_ values for hydroxyl radical and superoxide radical were 0.27 and 0.54 mg/mL, respectively. Lipid peroxidation inhibition rate was 47.78 ± 0.91 %. The DNA protection rate was 52.19 ± 1.34 %.	Xu et al. (62)
	PBP	*In vitro*	H_2_O_2_ induced red blood cells	0.2, 0.4, 0.8, 1.0, 2.0, and 4.0 mg/mL	The inhibition rate of hemolysis was 60.58 %±0.52 %. GSH contents ↑; MDA contents ↓;	Yang et al. (50)
	U-300	*In vitro*	H_2_O_2_ induced red blood cells	0.2, 0.4, 0.8, 1.0, 2.0, and 4.0 mg/mL	The inhibition rate of hemolysis was 67.73 %±0.18 %. GSH contents ↑; MDA contents ↓;	Yang et al. (50)
	U-500	*In vitro*	H_2_O_2_ induced red blood cells	0.2, 0.4, 0.8, 1.0, 2.0, and 4.0 mg/mL	The inhibition rate of hemolysis was 70.22 %±0.62 %. GSH contents ↑; MDA contents ↓;	Yang et al. (50)
	U-700	*In vitro*	H_2_O_2_ induced red blood cells	0.2, 0.4, 0.8, 1.0, 2.0, and 4.0 mg/mL	The inhibition rate of hemolysis was 76.24 %±0.71 %. GSH contents ↑; MDA contents ↓;	Yang et al. (50)
	PRTP	*In vitro*	DPPH, hydroxyl, and ABTS radicals	0.2, 0.4, 0.6, 0.8, and 1.0 mg/mL	The IC_50_ of DPPH radical, hydroxyl radical, and ABTS radical were 0.102, 0.976, and 1.189 mg/mL, respectively.	Yang et al. (59)
	PRBP	*In vitro*	DPPH, hydroxyl, and ABTS radicals	0.2, 0.4, 0.6, 0.8, and 1.0 mg/mL	The IC_50_ of DPPH radical, hydroxyl radical, and ABTS radical were 0.857, 0.874, and 0.842 mg/mL, respectively.	Yang et al. (59)
	BBP	*In vitro*	DPPH radicals	NM	DPPH free radical scavenging rate was 37.29 %.	Konrade et al. (42)
	BCP	*In vitro*	DPPH, hydroxyl, and superoxide anion radicals, reducing power, ferrous ion-chelating capacity	0.2, 0.4, 0.6, 0.8, 1.0, and 1.2 mg/mL	The IC_50_ values of DPPH and superoxide anion radical were 0.81 and 0.46 mg/mL, respectively. The maximum clearance rates were 71.50 ± 0.85 %, 42.73 ± 0.67 %, and 73.50 ± 2.46 %, respectively. The reducing ability was positively correlated with the increase of concentration. The chelating ability was 16.25 %.	Xu et al. (54)
	SBCP-1	*In vitro*	DPPH, hydroxyl, and superoxide anion radicals, reducing power, ferrous ion-chelating capacity	0.2, 0.4, 0.6, 0.8, 1.0, and 1.2 mg/mL	The IC_50_ values of DPPH and superoxide anion radical were 0.33 and 0.23 mg/mL, respectively. The maximum clearance rates were 89.60 ± 1.80 %, 95.71 ± 1.99 %, and 91.82 ± 1.84 %, respectively. The reducing ability was positively correlated with the increase of concentration. The chelating ability was 35.71 %.	Xu et al. (54)
	SBCP-2	*In vitro*	DPPH, hydroxyl, and superoxide anion radicals, reducing power, ferrous ion-chelating capacity	0.2, 0.4, 0.6, 0.8, 1.0, and 1.2 mg/mL	The IC_50_ values of DPPH and superoxide anion radical were 0.46 and 0.34 mg/mL, respectively. The maximum clearance rates were 85.37 ± 1.27 %, 62.56 ± 5.03 %, and 89.62 ± 2.35 %, respectively. The reducing ability was positively correlated with the increase of concentration. The chelating ability was 23.31 %.	Xu et al. (54)
	SBCP-3	*In vitro*	DPPH, hydroxyl, and superoxide anion radicals, reducing power, ferrous ion-chelating capacity	0.2, 0.4, 0.6, 0.8, 1.0, and 1.2 mg/mL	The IC_50_ values of DPPH and superoxide anion radical were 1.38 and 0.44 mg/mL, respectively. The maximum clearance rates were 45.73 ± 0.66 %, 90.66 ± 1.37 %, and 83.49 ± 2.99 %, respectively. The reducing ability was positively correlated with the increase of concentration. The chelating ability was 18.87 %.	Xu et al. (54)
	RNP	*In vitro*	Hydroxyl and superoxide anion radicals, lipid peroxidation inhibition	0.2~1.2 mg/mL	The maximum clearance rates were 69.09 ± 2.51 % and 52.58 ± 2.44 %, respectively. The inhibition rate of lipid peroxidation activity was 9.91 ± 1.37 %	Duan et al. (18)
	CRNP-1	*In vitro*	Hydroxyl and superoxide anion radicals, lipid peroxidation inhibition	0.2~1.2 mg/mL	The maximum clearance rates were 70.69 ± 2.33 % and 63.63 ± 1.34 %, respectively. The inhibition rate of lipid peroxidation activity was 36.92 ± 0.71 %	Duan et al. (18)
	CRNP-2	*In vitro*	Hydroxyl and superoxide anion radicals, lipid peroxidation inhibition	0.2~1.2 mg/mL	The maximum clearance rates were 76.69 ± 1.69 % and 66.39 ± 1.09 %, respectively. The inhibition rate of lipid peroxidation activity was 45.46 ± 1.75 %	Duan et al. (18)
	CRNP-3	*In vitro*	Hydroxyl and superoxide anion radicals, lipid peroxidation inhibition	0.2~1.2 mg/mL	The maximum clearance rates were 78.57 ± 0.38 % and 76.03 ± 1.12 %, respectively. The inhibition rate of lipid peroxidation activity was 48.03 ± 0.26 %	Duan et al. (18)
	RNP	*In vitro*	AAPH and H_2_O_2_-induced hemolysis of red blood cells	0~4 mg/mL for 2 h	The inhibition rates of hemolysis were 48.07 ± 0.17 % and 50.95 ± 0.76 %, respectively. MDA level ↓; GSH level ↑;	Duan et al. (18)
	CRNP-1	*In vitro*	AAPH and H_2_O_2_-induced hemolysis of red blood cells	0~4 mg/mL for 2 h	The inhibition rates of hemolysis were 53.13 ± 0.41 % and 54.75 ± 0.27 %, respectively. MDA level ↓; GSH level ↑;	Duan et al. (18)
	CRNP-2	*In vitro*	AAPH and H_2_O_2_-induced hemolysis of red blood cells	0~4 mg/mL for 2 h	The inhibition rates of hemolysis were 57.99 ± 0.94 % and 57.12 ± 0.79 %, respectively. MDA level ↓; GSH level ↑;	Duan et al. (18)
	CRNP-2	*In vitro*	AAPH and H_2_O_2_-induced hemolysis of red blood cells	0~4 mg/mL for 2 h	The inhibition rates of hemolysis were 61.74 ± 0.94 % and 63.69 ± 0.94 %, respectively. MDA level ↓; GSH level ↑;	Duan et al. (18)
	RNLP I	*In vitro*	DPPH, hydroxyl, and superoxide anion radicals, reducing power	0.2~1.2 mg/mL, 0.2~3.5 mg/mL	The IC_50_ values of DPPH, hydroxyl free, and superoxide anion radical were 0.20, 1.74, and 0.34 mg/mL, respectively. The reduction ability is 0.884.	Xu et al. (44)
	BCP	*In vitro*	DPPH, hydroxyl radicals, reducing power	0.1 mg/mL	The maximum clearance rates were 43.09 % and 76.11 %, respectively. The reduction ability is 0.799.	Wang et al. (123)
	BCP	*In vitro*	DPPH, hydroxyl, and superoxide anion radicals	0.20, 0.40, 0.60, 0.80, and 1.00 mg/mL	The IC_50_ values of hydroxyl free and superoxide anion radical were 0.976 and 0.393 mg/mL, respectively. The maximum scavenging rate of DPPH free radical was 46.2%.	Yu et al. (83)
	BCP	*In vitro*	DPPH, hydroxyl, and superoxide anion radicals	0.20, 0.40, 0.60, 0.80, 1.00, and 1.2 mg/mL	The IC_50_ values of DPPH, hydroxyl free, and superoxide anion radical were 0.22, 0.33, and 0.64 mg/mL, respectively.	Xu et al. (63)
	BCP-1	*In vitro*	DPPH, hydroxyl, and superoxide anion radicals	0.20, 0.40, 0.60, 0.80, 1.00, and 1.2 mg/mL	The maximum clearance rates were 95.81 %, 57.66 %, and 71.05 %, respectively.	Xu et al. (55)
	BP	*In vitro*	DPPH, hydroxyl, and superoxide anion radicals	0.2~1.0 mg/mL	The maximum clearance rates were 88.79 ± 0.65 %, 32.58 ± 0.23 %, and 57.56 ± 0.66 %, respectively.	Xu et al. (43)
	ABP-1	*In vitro*	DPPH, hydroxyl, and superoxide anion radicals	0.2~1.0 mg/mL	The maximum clearance rates were 89.96 ± 0.73 %, 46.18 ± 0.53 %, and 61.79 ± 0.48 %, respectively.	Xu et al. (43)
	ABP-2	*In vitro*	DPPH, hydroxyl, and superoxide anion radicals	0.2~1.0 mg/mL	The maximum clearance rates were 90.35 ± 0.33 %, 55.10 ± 0.36 %, and 64.11 ± 0.33 %, respectively.	Xu et al. (43)
	ABP-3	*In vitro*	DPPH, hydroxyl, and superoxide anion radicals	0.2~1.0 mg/mL	The maximum clearance rates were 90.88 ± 0.32 %, 61.76 ± 0.69 %, and 77.45 ± 0.71 %, respectively.	Xu et al. (43)
Anti-diabetic activity						
	PRSP-1	*In vitro*	α-amylase and α-glucosidase	0.4~6.0 mg/mL	The IC_50_ values of α-amylase and α-glucosidase were 5.52 and 3.49 mg/mL, respectively.	Zhao et al. (53)
	PRSP-2	*In vitro*	α-amylase and α-glucosidase	0.4~6.0 mg/mL	The IC_50_ values of α-amylase and α-glucosidase were 4.47 and 3.28 mg/mL, respectively.	Zhao et al. (53)
	PRSP-3	*In vitro*	α-amylase and α-glucosidase	0.4~6.0 mg/mL	The IC_50_ values of α-amylase and α-glucosidase were 3.93 and 2.75 mg/mL, respectively.	Zhao et al. (53)
	PRCP	*In vitro*	α-amylase and α-glucosidase	0.4~6.0 mg/mL	The IC_50_ values of α-amylase and α-glucosidase were 6.26 and 4.23 mg/mL, respectively.	Zhao et al. (53)
	BCP-1	*In vitro*	BSA/glucose model	0.025, 0.05, 0.10, 0.15, and 0.20 mg/mL	Amadori products ↓; dicarbonyl compounds ↓; non-enzymatic glycation of proteins ↓;	Xu et al. (51)
	DBCP-1	*In vitro*	α-amylase and α-glucosidase	0.1~4.0 mg/mL	The IC_50_ values of α-amylase and α-glucosidase were 3.50 and 0.697 mg/mL, respectively.	Xu et al. (41)
	DBCP-2	*In vitro*	α-amylase and α-glucosidase	0.1~4.0 mg/mL	The IC_50_ values of α-amylase and α-glucosidase were 0.822 and 0.286 mg/mL, respectively.	Xu et al. (41)
	RP-SeNPs-1	*In vitro*	BSA/glucose model and α-glucosidase	0.10 and 0.50 mg/mL, 4~6.0 mg/mL	Amadori products ↓; dicarbonyl compounds ↓; AGEs ↓; The IC_50_ values of α-glucosidase was 2.142 mg/mL.	Zhao et al. (80)
	RP-SeNPs-2	*In vitro*	BSA/glucose model and α-glucosidase	0.10 and 0.50 mg/mL, 4~6.0 mg/mL	Amadori products ↓; dicarbonyl compounds ↓; AGEs ↓; The IC_50_ values of α-glucosidase was 2.561 mg/mL.	Zhao et al. (80)
	RP-SeNPs-3	*In vitro*	BSA/glucose model and α-glucosidase	0.10 and 0.50 mg/mL, 4~6.0 mg/mL	Amadori products ↓; dicarbonyl compounds ↓; AGEs ↓; The IC_50_ values of α-glucosidase was 2.760 mg/mL.	Zhao et al. (80)
	BCP	*In vitro*	α-amylase and α-glucosidase	0.1, 0.2, 0.4, 0.6, 0.8, 1.0, 2.0, and 4.0 mg/mL	It has a certain inhibition rate on α-amylase and α-glucosidase.	Xu et al. (62)
	U-400	*In vitro*	α-amylase and α-glucosidase	0.1, 0.2, 0.4, 0.6, 0.8, 1.0, 2.0, and 4.0 mg/mL	The inhibition rates of α-amylase and α-glucosidase were 61.49 ± 0.66 % and 58.96 ± 1.91 %, respectively.	Xu et al. (62)
	U-600	*In vitro*	α-amylase and α-glucosidase	0.1, 0.2, 0.4, 0.6, 0.8, 1.0, 2.0, and 4.0 mg/mL	The inhibition rates of α-amylase and α-glucosidase were 5.98 ± 0.77 % and 69.92 ± 0.93 %, respectively.	Xu et al. (62)
	BCP	*In vitro*	α-amylase	0.2, 0.4, 0.6, 0.8, 1.0, and 1.2 mg/mL	The inhibition rate of α-amylase was 25.55 ± 0.59 %.	Xu et al. (54)
	SBCP-1	*In vitro*	α-amylase	0.2, 0.4, 0.6, 0.8, 1.0, and 1.2 mg/mL	The inhibition rate of α-amylase was 53.81 ± 1.78 %.	Xu et al. (54)
	SBCP-2	*In vitro*	α-amylase	0.2, 0.4, 0.6, 0.8, 1.0, and 1.2 mg/mL	The inhibition rate of α-amylase was 47.91 ± 2.27 %.	Xu et al. (54)
	SBCP-3	*In vitro*	α-amylase	0.2, 0.4, 0.6, 0.8, 1.0, and 1.2 mg/mL	The inhibition rate of α-amylase was 45.57 ± 1.56 %.	Xu et al. (54)
	BCP I	*In vitro*	α-amylase	0.2, 0.4, 0.6, 0.80, 1.0, 2.0, 3.0, and 4.0 mg/mL	The inhibition rate of α-amylase was 26.44 ± 0.12 %.	Xu et al. (85)
	BCP	*In vitro*	α-amylase	0.0~1.2 mg/mL	The inhibition rate of α-amylase was 36.67 ± 1.10 %.	Xu et al. (63)
	BP	*In vitro*	α-amylase and α-glucosidase	0.2~1.0 mg/mL	The inhibition rates of α-amylase and α-glucosidase were 64.99 ± 1.72 % and 43.77 ± 0.22 %, respectively.	Xu et al. (43)
	ABP-1	*In vitro*	α-amylase and α-glucosidase	0.2~1.0 mg/mL	The inhibition rates of α-amylase and α-glucosidase were 72.79 ± 1.58 % and 65.21 ± 0.24 %, respectively.	Xu et al. (43)
	ABP-2	*In vitro*	α-amylase and α-glucosidase	0.2~1.0 mg/mL	The inhibition rates of α-amylase and α-glucosidase were 72.39 ± 1.62 % and 72.66 ± 0.06 %, respectively.	Xu et al. (43)
	ABP-3	*In vitro*	α-amylase and α-glucosidase	0.2~1.0 mg/mL	The inhibition rates of α-amylase and α-glucosidase were 90.16 ± 1.11 % and 83.22 ± 0.09 %, respectively.	Xu et al. (43)
	BCP	*In vivo*	High-fat diet/streptozotocin-induced diabetic mice	100 and 300 mg/kg *i.g*., once daily, for 10 weeks	FBG, Cr, BUN, T-CHO, TG, TNF-α, IL-1β, IL-6 levels ↓; Perirenal fat index ↓; Kidney index ↓; SOD activity ↑; MDA content ↓; *Akkermansia muciniphila* abundance ↑; Firmicutes/Bacteroidetes ratio ↓; GPBAR1, Nrf2, and HO-1 protein expression↑; P-IκB, P-NF-κB p65, TNF-α, IL-6, NLRP3, Caspase-1 p20, IL-1β, TGF-β1, and P-Smad3 protein expression↓;	Zhu et al. (86)
Immunoregulation activities						
	CAPS	*In vitro*	Bone marrow-derived dendritic cells	10 and 100 μg/mL for 24 h	TNF-α level ↑; TNF-α, IL-1β, and IL-12p40 mRNA expression ↑;	Ashigai et al. (47)
	CAPS-l.m.	*In vitro*	ICR female mice macrophages	0.1, 10, 100, and 1000 μg/mL	TNF-α, IL-1β, IL-12p70, and NO levels ↑;	Takata et al. (64)
	CAPS-h.m.	*In vitro*	ICR female mice macrophages	0.1, 10, 100, and 1000 μg/mL	TNF-α, IL-1β, IL-12p70, and NO levels ↑;	Takata et al. (64)
	CAPS	*In vivo*	ICR female mice splenocytes	10 mL/kg *i.g*., once daily, for 21 d	IL-2, IL-10, and IFN-γ levels ↑; IL-4 level ↓;	Takata et al. (64)
	CAPS	*In vitro*	RAW 264.7 cells	200 μg/mL	TNF-α level ↑;	Takata et al. (89)
	BCP	*In vivo*	Cyclophosphamide-induced BALB/c mice	0.4, 0.8, and 1.6 mg/g *i.p*., once daily, for 4 d	Thymus index, spleen index, mononuclear macrophage function ↑; IL-2, PFC, and HC50 level ↑;	Li et al. (88)
Anti-inflammatory activities						
	CAPS	*In vivo*	UV-irradiated mice	0.2 % and 1 %, *i.g*., once daily, for 18 d	Water loss ↓; IL-6 and MMP-13 mRNA expression ↓;	Ashigai et al. (48)
	15.6-7.4,-26690pt	CAPS	*In vivo*	NC/Nga mice	12 and 60 mg/kg, *i.g*., once daily, for 28 d	Atopic dermatitis scores ↓; IgE level ↓; IFN-γ, IL-12p40, and TNF-α mRNA expression ↑; IL-4 mRNA expression ↓; Number of mast cells ↓;	Ashigai et al. (49)
Anti-tumor activity						
	CAPS	*In vivo*	Mouse Ehrlich solid tumor model	10 mL/kg *i.g*., once daily, for 21 d	The tumor inhibition rate was 51 %.	Takata et al. (64)
	15.6-7.4,-1690pt	CAPS	*In vivo*	Mouse Ehrlich solid tumor model	10 mL/kg *i.g*., once daily, for 21 d	Tumor mass was significantly reduced.	Takata et al. (89)
Anti-bacterial adhesion activity						
	F2	*In vitro*	*Helicobacter pylori* and Le^b^-gastric epithelial cells	1 and 4 mg/mL	The inhibition of bacterial adhesion was about 25 % and 40 %.	Sivam et al. (95)
	RPS	*In vitro*	*Helicobacter pylori* and human gastric mucosa	0.1, 0.5, and 1 mg/mL	It can significantly inhibit the adhesion of human gastric mucosa in a concentration-dependent manner.	Lengsfeld et al. (96)
Anti-aging activity						
	BCP	*In vitro*	Non-enzymatic glycosylation system	0.05, 0.1, 0.15, and 0.20 mg/mL for 14 d	The inhibition rates of the three stages of non-enzymatic glycosylation reaction were 34.78 %, 30.80 % and 43.14 %, respectively.	Yu et al. (83)
	BCP	*In vitro*	Non-enzymatic glycosylation system	0.05, 0.20, and 0.40 mg/mL	The inhibition rates of the three stages of non-enzymatic glycosylation reaction were 42.82 %, 73.24 % and 76.84 %, respectively.	Xu et al. (63)
Anti-gout activities						
	PRNP-1	*In vitro*	Xanthine oxidase	0, 1.25, 2.5, and 5.0 mg/mL	It showed a strong inhibitory effect on XOD, and the inhibition rate was 53.64 ± 2.87 %.	Huang et al. (58)
	PRNP-2	*In vitro*	Xanthine oxidase	0, 1.25, 2.5, and 5.0 mg/mL	It showed a strong inhibitory effect on XOD, and the inhibition rate was 64.75 ± 3.16 %.	Huang et al. (58)
	PRNP-1	*In vivo*	Potassium oxide and xanthine induced hyperuricemia in mice	250 and 500 mg/kg, *i.g*., once daily, for 15 d	UA and Cr content ↓; XOD activity ↓;	Huang et al. (58)
	PRNP-2	*In vivo*	Potassium oxide and xanthine induced hyperuricemia in mice	250 and 500 mg/kg, *i.g*., once daily, for 15 d	The activity of PRNP-2 was better than that of PRNP-1; UA and Cr content ↓; XOD activity ↓;	Huang et al. (58)
Skin repair activity						
	F2	*In vitro*	Primary normal human fibroblasts from human skin and HaCaT keratinocytes	1, 10, and 100 μg/mL for 48 h	Cell viability ↑; dehydrogenase activity ↑; catabolic enzymes, DNA repair, extracellular matrix proteins, and signal transduction factors ↑;	Zippel et al. (71)
Acetylcholinesterase inhibitory activity						
	PRTP	*In vitro*	AChE	1, 2, 3, 4, and 5 mg/mL	The inhibition rate of AChE was 30.13 ± 0.87 %.	Yang et al. (59)
	PRBP	*In vitro*	AChE	1, 2, 3, 4, and 5 mg/mL	The inhibition rate of AChE was 34.61 ± 1.04 %.	Yang et al. (59)

PRNP-1, PRNP-2, PRCP, BCP-1, DBCP-1, DBCP-2, CAPS, BCP, U-400, U-600, CAPS, PBP, U-300, U-500, U-700, PRTP, PRBP, RNP, RNLP I, BCP I, CAPS-l.m., CAPS-h.m., polysaccharide from *R. nigrum* fruits; PRSP-1, PRSP-2, PRSP-3, RP-SeNPs-1, RP-SeNPs-2, RP-SeNPs-3, selenized polysaccharides from *R. nigrum* fruits; F2, RPS polysaccharides from *R. nigrum* seeds; BBP, pectin from *R. nigrum*; SBCP-1, SBCP-2, SBCP-3, sulfated polysaccharides from *R. nigrum* fruits; CRNP-1, CRNP-2, CRNP-2 carboxymethylated polysaccharides from *R. nigrum* fruits; ABP-1, ABP-2, ABP-3, acetylated polysaccharides from *R. nigrum* fruits.

### 5.1 Anti-oxidant activity

Nowadays, anti-oxidation has become a common keyword in the fields of food, medicine and cosmetics. Plant polysaccharides are expected to replace synthetic anti-oxidants because of their diverse structures, green sources and low cytotoxicity (81, 82). In recent years, the anti-oxidant research of RNPs has increased exponentially, but there is a lack of systematic review. Based on this, we systematically reviewed the research on the anti-oxidant activity of RNPs, and pointed out the current methodological bottlenecks and future research directions.

The extraction process is the primary step to determine the primary structure and anti-oxidant activity of RNPs. The following research systematically compared the effects of different extraction strategies such as microwave, pressurized water and ultrasound on its free radical scavenging ability, providing a basis for subsequent structural optimization. Yang et al. (59) isolated two polysaccharides, PRTP and PRBP, from *R. nigrum* using a microwave-assisted dual-phase aqueous system. PRTP exhibited IC_50_ values of 0.102, 0.976, and 1.189 mg/mL against DPPH, hydroxyl, and ABTS^+^ radicals, respectively, while PRBP exhibited IC_50_ values of 0.857, 0.874, and 0.842 mg/mL, respectively. Both polysaccharides demonstrated high-efficiency scavenging of multiple free radicals at low concentrations. The polysaccharide RNLP I extracted under pressurized water exhibited dose-dependent inhibition of DPPH, hydroxyl, and superoxide anion radicals within the range of 0.2~1.0 mg/mL. It demonstrated a maximum DPPH scavenging rate of 93.79 % and a reduction capacity as high as 0.884 at 3.5 mg/mL, significantly surpassing common plant polysaccharides (44). The polysaccharide BCP extracted by ultrasound had the strongest scavenging ability to superoxide anion, and the IC_50_ was 0.393 mg/mL. The hydroxyl radical IC_50_ is 0.976 mg/mL. The DPPH free radical scavenging rate was 46.2 % at 1 mg/mL, which was comparable to that of Vc (83). The IC_50_ of BCP extracted by the same method was 0.22 mg/mL (DPPH), 1.11 mg/mL (hydroxyl), and 0.64 mg/mL (superoxide anion) in 0.2~1.2 mg/mL, and the DPPH free radical scavenging peak was 91 %, which was close to Vc (63). In addition, among the BCP-1 polysaccharides obtained by ultrasonic-gradient ethanol precipitation, the highest scavenging rates of BCP-1-II on DPPH, hydroxyl and superoxide anion radicals were 95.81 %, 57.66 %, and 71.05 %, respectively, in a concentration-dependent manner (55). In summary, ultrasonic, microwave or pressurized water extraction can significantly retain or enhance the free radical scavenging ability of RNPs, and further grading and purification can obtain homogeneous components with higher activity.

Although optimizing the extraction conditions can improve the activity of polysaccharides, their inherent structure may still limit their anti-oxidant potential. Chemical modification provides an effective means to break through the bottleneck of activity by introducing functional groups. The following studies have verified the enhancement effect of sulfation, carboxymethylation and acetylation on the anti-oxidant properties of RNPs. Xu et al. (54) successfully prepared three sulfated derivatives (SBCPs) with different degrees of substitution (DS) by sulfated modification of BCP, and systematically evaluated their anti-oxidant activity *in vitro*. The results showed that SBCPs were significantly superior to unmodified BCPs in DPPH, hydroxyl radical and superoxide anion radical scavenging ability, and the activity increased with the increase of DS. Among them, SBCP-1 (DS = 1.28) showed the strongest anti-oxidant performance. At a concentration of 1.2 mg/mL, its hydroxyl radical scavenging rate reached 95.71 ± 1.99 %, which was higher than that of the positive control Vc (90.66 ± 1.37 %) under the same experimental conditions. The IC_50_ value of SBCP-1 for hydroxyl radicals was also lower than that of Vc, indicating superior radical-scavenging capacity. In addition, SBCPs also showed good reducing power and Fe^2+^ chelating ability, which further confirmed that they played an anti-oxidant role through hydrogen atom supply and metal ion chelation mechanism. Duan et al. (18) prepared carboxymethylated polysaccharides (CRNPs) by carboxymethylation of natural polysaccharides (RNP) extracted from *R. nigrum* fruit. It was found that CRNPs showed stronger ability to scavenge hydroxyl and superoxide anion radicals, and their scavenging activity increased with the increase of DS, among which CRNP-3 with high degree of substitution had the strongest activity. In addition, CRNPs also showed a dose-dependent protective effect on inhibiting lipid peroxidation and protecting red blood cells from hemolysis induced by AAPH and H_2_O_2_, significantly reducing MDA production and increasing reduced GSH levels, indicating that it can exert anti-oxidant effects by reducing oxidative stress and maintaining cell redox homeostasis. Xu et al. (43) prepared three kinds of acetylated polysaccharides (ABP-1, ABP-2, ABP-3) with different DS by acetylation modification of *R. nigrum* fruit polysaccharide (BP). The DPPH, hydroxyl, and superoxide anion radical scavenging ability of acetylated polysaccharides were significantly higher than that of unmodified BP, and increased in a degree of substitution dependent manner. The order of activity was: BP < ABP-1 < ABP-2 < ABP-3 < positive control V_C_. The maximum scavenging rates of DPPH, hydroxyl, and superoxide anion radical by ABP-3 at 1.0 g/L were 90.88 %, 61.76 %, and 77.45 %, respectively, indicating that acetylation modification could significantly improve the anti-oxidant capacity of BP, especially in scavenging hydroxyl radicals and superoxide anions. This chemical modification improves the hydrogen atom supply and metal chelating ability by introducing negative charge or hydrophobic groups, and achieves a leap-forward improvement in anti-oxidant efficiency.

In addition to chemical modification, physical degradation is considered to be a green strategy to enhance the biological activity and bioavailability of polysaccharides. The following studies reduced the molecular weight by physical degradation treatment, and systematically evaluated the anti-oxidant performance of the degradation products *in vitro* and in cell models. Xu et al. (41) systematically compared the anti-oxidant spectra of BCP and its Fe^2+^-H_2_O_2_ degradation components DBCP-1 and DBCP-2. The results showed that DBCP-2 with the lowest molecular weight was the most prominent: DPPH scavenging IC_50_ decreased from 0.72 mg/mL of BCP to 0.40 mg/mL; at the dose of 1.2 mg/mL, the scavenging rates of hydroxyl and superoxide anion radical were 93.2 % and 62.1 %, respectively, which were significantly better than those of the parent polysaccharide. Its reducing power reached 0.414, and the chelating rate of Fe^2+^ also increased to 23.3 %. It can be seen that controlled degradation not only reduces the molecular weight, but also comprehensively enhances the anti-oxidant potential of BCP by exposing active groups. The anti-oxidant activity of U-400 and U-600 obtained by ultrasonic degradation of BCP was positively correlated with concentration-effect, and the lower the molecular weight, the more significant the advantage. The IC_50_ of hydroxyl radical and superoxide anion radical scavenging decreased from 0.40 and 1.20 mg/mL of BCP to 0.27 and 0.54 mg/mL, respectively, and the order of activity was U-600 > U-400 > BCP. At 1.2 mg/mL, the inhibition rate of U-600 on lipid peroxidation of lecithin induced by Fe^2+^-V_C_ was 47.8 %, which was 35 % higher than that of the original polysaccharide. The protection rate against hydroxyl radical-mediated herring sperm DNA damage increased to 52.2 % at 4.0 mg/mL, which was twice that of BCP. It can be seen that ultrasonic shearing can fully amplify the free radical scavenging, lipid protection and DNA damage protection ability of BCP by reducing molecular weight and exposing active sites (62). Yang et al. (50) confirmed that low molecular weight black currant polysaccharides U-300, U-500, and U-700 obtained by ultrasonic degradation could dose-dependently inhibit hemolysis induced by H_2_O_2_, and the highest inhibition rates were 67.7 %, 70.2 %, and 76.2 %, respectively, which were superior to the original polysaccharides and Trolox. At the same time, it significantly increased intracellular GSH, reduced MDA, and alleviated membrane oxidative damage. The anti-oxidant activity increased with the decrease of molecular weight. Degradation treatment significantly enhanced anti-oxidant performance by reducing molecular weight, increasing terminal reducing groups, increasing the probability of collision between polysaccharides and free radicals and cell membrane permeability.

In addition to the extraction, modification and degradation strategies, the anti-oxidant potential of different parts and purified components of *R. nigrum* has also attracted attention. Konrade et al. (42) reported that the DPPH scavenging rate of *R. nigrum* pectin was 37.29 %, ranking first in the genus *Ribes*. RNPs have shown great potential to replace synthetic anti-oxidants, and RNPs prepared by various methods show significant activity. However, most of the existing studies only focus on the activity evaluation by *in vitro* chemical methods, and cell-animal-clinical evidence is scarce. At the same time, the relationship between extraction-modification-degradation parameters and product structure-activity is still empirical optimization, lacking multi-scale model guidance. In the future, a unified extraction-modification-degradation process coding and structure-activity database will be established, and a process-structure-activity prediction model will be established in combination with machine learning to achieve rational design. The animal model of oxidative stress, PK-PD and intestinal flora metabolomics were further constructed to determine the minimum effective dose, bioavailability and functional metabolites.

### 5.2 Anti-diabetic activity

In the face of the global trend of high incidence of diabetes, exploring safe and efficient natural carbohydrate regulators has become a core issue in the field of functional foods (84). RNPs have recently been given more hypoglycemic functions due to their good biocompatibility and modifiability. Zhao et al. (53) reported a facile selenylation strategy that converts native *R. nigrum* polysaccharide (PRCP) into selenised derivatives (PRSPs). The introduced selenate groups not only reduce molecular weight but also markedly enhance water solubility and thermal stability. Among the series, PRSP-3 exhibited the strongest dose-dependent inhibition against α-amylase and α-glucosidase, with IC_50_ values approaching those of acarbose. Kinetic analyses revealed a reversible competitive mechanism: PRSPs occupy the catalytic pocket of the enzymes, while fluorescence quenching and thermodynamic studies indicate that hydrophobic interactions and hydrogen bonds stabilize the enzyme-PRSP complex, trigger micro-environmental conformational changes, and ultimately lower substrate affinity—thus retarding carbohydrate hydrolysis and post-prandial hyperglycaemia. Building on this concept, Zhao et al. (80) further fabricated polysaccharide-stabilized selenium nanoparticles (RP-SeNPs) via self-assembly. The smallest-sized fraction (RP-SeNPs-1) displayed superior, dose-dependent suppression of all three stages of protein glycation (Amadori products, dicarbonyl intermediates, and AGEs). In parallel, RP-SeNPs-1 outperformed bare SeNPs in inhibiting α-glucosidase, showing a lower IC_50_ and the highest binding constant in competitive kinetic assays. Isothermal titration calorimetry and fluorescence quenching demonstrated that hydrogen-bond formation is the dominant force driving the reversible competitive inhibition, underscoring that nano-architecturing amplifies both enzyme-binding affinity and anti-glycation efficacy. Collectively, these studies establish that selenylation or nano-encapsulation of RNPs generates bifunctional constructs capable of concurrently slowing starch digestion and blocking AGE formation. The dual modulation relies on size reduction, increased hydrogen-bond donor capacity, and strengthened enzyme-binding affinity—offering a promising blueprint for developing next-generation, food-grade hypoglycaemic ingredients.

In addition, chemically derivatized polysaccharides also showed hypoglycemic activity. Xu et al. (54) prepared a series of sulfated black currant polysaccharides (SBCPs) with varying DS. All derivatives exhibited concentration-dependent suppression of α-amylase, with SBCP-1 (DS = 1.28) reaching 53.81 % inhibition at 1.2 mg/mL—approximately double that of the native BCP (25.55 %), underscoring that sulfate grafting is a straightforward strategy to amplify hypoglycaemic potential. In parallel, acetylated counterparts (ABP-1, ABP-2, and ABP-3) displayed a DS-dependent enhancement toward both α-amylase and α-glucosidase. The highest substituted ABP-3 achieved maximal inhibitions of 90.16 % and 83.22 %, respectively, with IC_50_ values (0.071 g/L for α-amylase, 0.011 g/L for α-glucosidase) approaching those of acarbose (43). Collectively, these data demonstrate that either anionic (-${\text{SO}}_{3}^{-}$) or hydrophobic (-COCH_3_) functionalization consistently strengthens enzyme binding, providing a robust, tunable route for designing next-generation, carbohydrate-based glycaemic regulators.

Physically degraded polysaccharides also showed excellent hypoglycemic potential. Fe^2+^-H_2_O_2_-mediated degradation of BCP generated two depolymerised fractions, DBCP-1 and DBCP-2, with markedly enhanced inhibitory activity against α-amylase and α-glucosidase. DBCP-2 exhibited an IC_50_ of 0.822 mg/mL toward α-amylase and achieved 67.98±0.62 % suppression of α-glucosidase at the tested concentration, both superior to native BCP. The lowered molecular weight and increased reducing-end exposure were identified as key factors delaying starch hydrolysis and glucose absorption, underscoring the value of controlled oxidative cleavage in producing potent, food-grade hypoglycaemic agents (41). Xu et al. (62) further demonstrated that ultrasonic degradation is an eco-friendly and scalable alternative. The resulting fractions U-400 and U-600 displayed dose-dependent inhibition across 0.1~4.0 mg/mL with U-600 exhibiting the highest efficacy: 76 % inhibition of α-amylase and 70 % inhibition of α-glucosidase at 4.0 mg/mL, with IC_50_ values (0.98 and 1.15 mg/mL, respectively) significantly lower than that of acarbose. Collectively, these findings confirm that both oxidative and ultrasonic depolymerisation can markedly amplify the enzyme-inhibitory capacity of blackcurrant polysaccharides, offering practical pathways for developing next-generation, natural glycaemic regulators.

Extraction technology as a critical lever for enhancing anti-diabetic activity. Employing an ultrasound-assisted composite enzymolysis protocol, Xu et al. (51) isolated a novel polysaccharide (BCP-1) from *R. nigrum* and evaluated its antiglycation potential in a BSA/glucose non-enzymatic glycation model. BCP-1 exerted superior suppression in the late phase of the Maillard reaction, inhibiting dicarbonyl compounds and advanced glycation end-products (AGEs) by 66.95 % and 67.15 %, respectively, while showing moderate restraint (37.15 %) against Amadori products. These findings highlight BCP1′s ability to sequester reactive carbonyl species and block AGE formation, positioning it as a promising functional ingredient for mitigating diabetic complications. Using the same ultrasound-combined enzymatic extraction, researchers obtained a distinct fraction (BCP I) that displayed dose-dependent inhibition of α-amylase (up to 26.44 ± 0.12 % at 4 mg/mL). Although the inhibitory potency is moderate, the result corroborates that integrative cell-wall disruption techniques can liberate bioactive polysaccharide segments with intrinsic enzyme-suppressing capacity (85). Collectively, these studies underscore that advanced extraction methodologies not only improve yield but also enrich specific structures responsible for antiglycation and α-amylase inhibition, thereby expanding the repertoire of RNPs-derived hypoglycaemic candidates.

While the aforementioned studies have firmly established the *in vitro* hypoglycemic potential of RNPs, *in vivo* evidence, particularly regarding their role in mitigating diabetic complications, has remained scarce. Addressing this gap, a recent study employing a high-fat diet/streptozotocin-induced diabetic mouse model demonstrated that intragastric administration of BCP (100 mg/kg and 300 mg/kg) for 10 weeks significantly improved glucose homeostasis and, notably, alleviated renal inflammation and fibrosis. Impressively, the renal protective effect of BCP was superior to that of metformin, the first-line antidiabetic drug. Mechanistic investigations using 16S rRNA sequencing revealed that BCP could reshape the intestinal microbiota structure, significantly enriching the population of *Akkermansia muciniphila*. The causal role of this bacterium was validated through fecal microbiota transplantation and exogenous *A. muciniphila* supplementation experiments, confirming it as a key effector for BCP's renoprotective activity. Further metabolomics analysis uncovered that *A. muciniphila* was strongly positively correlated with glycochenodeoxycholic acid (GUDCA), and both BCP and *A. muciniphila* treatment elevated serum GUDCA levels. In-depth mechanistic studies elucidated that GUDCA acts as an agonist of the GPBAR1 receptor, activating the renal GPBAR1 signaling pathway. This cascade concurrently inhibited the NF-κB/NLRP3 inflammasome-mediated inflammatory response, activated the Nrf2/HO-1 antioxidant pathway, and reduced renal fibrosis by suppressing the TGF-β1/Smad3 signaling. Direct supplementation with GUDCA recapitulated the beneficial effects, improving glucose metabolism and alleviating renal injury and inflammation. In summary, this work provides compelling evidence that BCP exerts its renoprotective effects in diabetes by modulating the gut microbiota-bile acid metabolism axis, offering a novel dietary intervention strategy for diabetic nephropathy (86).

In summary, RNPs have become the focus of research and development of natural carbohydrate-based blood glucose regulators due to their biocompatibility and modifiability. Current evidence robustly supports RNPs *in vitro* efficacy as enzyme inhibitors and anti-glycation agents. Furthermore, emerging *in vivo* studies have begun to unravel their sophisticated systemic mechanisms, such as the modulation of the gut-kidney axis, highlighting their potential in managing diabetic complications beyond simple glycemic control. However, the current research still lacks a certain basis. Most of the reports are *in vitro* studies and lack a complete evidence chain of structure-mechanism-dose-exposure-risk. At the same time, due to the lack of toxicological studies, it is difficult to ensure the safety of polysaccharide selenization and derivatization. Therefore, in the future, *in vivo* pharmacokinetic-pharmacodynamic models, chronic toxicity and metabolomics must be introduced to establish a rational design framework based on structural homogeneity and safety.

### 5.3 Immunoregulation activity

Immunomodulatory activity represents one of the most fundamental and extensively investigated pharmacological properties of natural polysaccharides. This property serves as a mechanistic foundation for their diverse health-promoting effects, including enhancement of anti-infective defense and anti-tumor immune responses (87). In line with this well-established paradigm, emerging evidence indicates that RNPs exhibit potent immunomodulatory activity, positioning them as promising multifunctional candidates for next-generation immunotherapeutic strategies. Some scholars have extracted an immunostimulatory active polysaccharide (CAPS) from *R. nigrum*. It was found that CAPS stimulated the production of TNF-α in a concentration-dependent manner *in vitro*, and CAPS also stimulated the gene expression of TNF-α, IL-1β, and IL-12p40. In addition, CAPS-induced TNF-α production was completely inhibited in TLR4–/– and MyD88–/– mouse models, indicating that CAPS activates DCs through the MyD88-dependent TLR4 signaling pathway. Further confirmed its activation of the immune system (47). In addition, some scholars further classified *R. nigrum* polysaccharide (CAPS) and found that the activity of low molecular weight fragment CAPS-l.m. stimulated thioglycolate-induced macrophage secretion of TNF-α, IL-1β, IL-12p70, and NO was significantly higher than that of high molecular weight CAPS-h.m. Subsequently, oral administration of CAPS could up-regulate IL-2, IL-10, and IFN-γ in spleen cells of Ehrlich cancer mice, and partially repair IL-4, suggesting that CAPS could enhance Th1 response and reverse tumor-related immune deviation, playing a two-way immune regulation (64). Li et al. (88) reported that BCP can resist cyclophosphamide-induced immunosuppression in a dose-dependent manner, restore thymus index and spleen index, and significantly increase macrophage phagocytosis rate and IL-2 level in medium and high doses. High-dose BCP can also stimulate antibody-producing cells and increase serum hemolysin HC50, and the effect is comparable to that of positive astragalus polysaccharides, confirming its immune reconstitution ability in a chemical immunocompromised model. The *R. nigrum* polysaccharide (CAPS) showed immunomodulatory activity *in vitro*. After enzymatic hydrolysis, although the TNF-α induction activity of RAW264.7 macrophages *in vitro* decreased slightly with the decrease of molecular weight, it still maintained a high level of immune stimulation, suggesting that enzymatic hydrolysis did not destroy its immune active epitope (89).

RNPs function as multifunctional immunomodulators by directly activating the MyD88-dependent TLR4 pathway in dendritic cells and macrophages. Beyond this direct immune cell activation, emerging evidence suggests that dietary polysaccharides, including RNPs, may also exert immunoregulatory effects indirectly through the gut microbiome axis. As recently articulated by Zhu et al. (90) in their precision medicine food intervention framework, the triad of metabolites (e.g., SCFAs), targeted microbial delivery, and AI-assisted profiling offers a powerful strategy to elucidate the complex interplay between food-derived polysaccharides and the host immune system. Future studies on RNPs should therefore integrate gut microbiota analysis, metabolomics, and computational modeling to construct a comprehensive ‘gut microbiome-immune axis' map. Nevertheless, the current body of evidence remains largely confined to *in vitro* assays or acute mouse models, with most studies utilizing crude or partially characterized fractions. Comprehensive data on structure-activity relationships, receptor binding kinetics, chronic toxicity, and human trials are still lacking. Until systematic investigations into activity, structure, and toxicology are completed, the promising prospects of RNPs as next-generation immunotherapies await full substantiation.

### 5.4 Anti-inflammatory activity

RNPs exhibit certain anti-inflammatory activity, which can reduce the inflammatory response of various diseases and play a therapeutic role. Some scholars have discussed the improvement effect of *R. nigrum* polysaccharide (CAPS) on skin dehydration induced by ultraviolet radiation. CAPS can significantly improve skin hydration and reduce transepidermal water loss in a dose-dependent manner. In addition, CAPS can also reduce the transcription levels of IL-6 and MMP in the skin, indicating that it improves skin hydration by inhibiting inflammatory response and collagen degradation (48). In addition, some scholars used the NC/Nga mouse model to explore the effect of CAPS on atopic dermatitis (AD). CAPS can effectively reduce serum IgE levels, inhibit the expression of Th2-type cytokine IL-4, and up-regulate the transcription of Th1-type cytokines IFN-γ and IL-12p40, thereby correcting the unbalanced Th1/Th2 immune response in AD model mice. In addition, CAPS significantly reduced the number of mast cell infiltration in skin tissue, alleviated epidermal hyperplasia and inflammatory response, and improved skin barrier function (49). The existing evidence consistently shows that RNPs exhibit significant anti-inflammatory effects in the skin barrier, providing a conceptual verification for the development of multifunctional skin care or AD adjuvants. However, the research still stays at the level of phenotypic observation and a small number of inflammatory indicators, and lacks active ingredient chain structure, receptor target, percutaneous penetration kinetics and long-term safety data. In the future, it is necessary to combine standardized polysaccharide grading, human-derived reconstructed epidermis and clinical randomized trials to confirm its efficacy and mechanism credibility.

### 5.5 Anti-tumor activity

Among the diverse pharmacological activities of plant polysaccharides, anti-tumor activity stands out as one of the most extensively studied and clinically promising fields, largely due to the ability of natural polysaccharides to safely suppress tumors by reshaping the immune microenvironment rather than exerting direct cytotoxicity. This feature has made them a research hotspot in adjuvant strategies for tumor immunotherapy (91, 92). In line with this trend, blackcurrant polysaccharides (RNPs) have also demonstrated significant anti-tumor activity in several well-documented studies. Some scholars found that in the mouse Ehrlich solid tumor model, the 21-day oral intervention of *R. nigrum* polysaccharide (CAPS) reduced tumor weight by 51 %, showing a similar tumor weight inhibition rate to that of OK-432 and adriamycin in the same experimental setting, but without obvious toxicity. In terms of mechanism, CAPS mainly relies on host-mediated immune activation rather than direct cytotoxicity. The anti-tumor effect is closely related to the rebalance of Th1/Th2 cytokine network in tumor macrophages and spleen cells (64). In addition, the anti-tumor activity of a pectin-type polysaccharide CAPS from *R. nigrum* was significantly enhanced in a molecular weight-dependent manner after moderate hydrolysis by Aspergillus β-galactosidase. When the average molecular weight decreased to about 19 kDa, the inhibitory effect of oral administration on Ehrlich ascites carcinoma in ICR mice reached the peak, and the tumor mass was significantly lower than that in PBS group. However, neither high molecular weight nor overhydrolyzed products had significant anti-tumor effect (89). In summary, these findings confirm that RNPs represent promising safe antitumor adjuvants whose efficacy depends on immune microenvironment modulation and optimal molecular weight. Future studies should elucidate the precise molecular mechanisms of RNPs within the tumor microenvironment. Concurrently, the antitumor efficacy and safety of RNPs should be validated across more diverse tumor models, and preclinical pharmacokinetic studies should be conducted to provide robust experimental evidence for developing RNP-based oral tumor immunotherapy adjuvants.

### 5.6 Anti-bacterial adhesion activity

The irreversible adhesion of *Helicobacter pylori* to gastric mucosa is the primary link of its colonization, persistent inflammation and gastric cancer (93). The non-lethal strategy with anti-adhesion as the core has become a new focus of disease treatment (94). Studies have found that RNPs exhibit certain anti-bacterial adhesion activity. Jutta et al. isolated and purified an arabinogalactan protein F2 from *R. nigrum* seeds. F2 inhibits the adhesion of *Helicobacter pylori* to Le∧b antigen and gastric epithelial cells by non-specifically binding to bacterial surface structure, blocking BabA and fibronectin-binding adhesin, and reducing the adhesion by 25 % and 40 % in tissue sections and AGS cell models, respectively. However, it had no effect on SabA-mediated sialic acid-Lewis∧x binding and hemagglutination activity, and did not induce changes in adhesion or virulence factor gene expression. The radioligand experiment further confirmed that F2 still binds to the BabA deletion strain, suggesting that its target is not single, and the mechanism is surface charge/steric hindrance-mediated broad-spectrum anti-adhesion (95). Some scholars have found that high molecular weight acidic polysaccharides (RPS) derived from *R. nigrum* seeds showed significant anti-*H.pylori* activity in an *in vitro* human gastric mucosal adhesion model. The polysaccharide inhibited the adhesion of *Helicobacter pylori* to gastric mucosa in a concentration-dependent manner by specifically acting on the adhesion factors on the surface of bacteria. It is worth noting that the polysaccharide has no growth inhibition or toxic effect on bacteria within the effective concentration range, and it is only effective for bacterial pretreatment and ineffective for mucosal pretreatment, suggesting that its mechanism of action is to block the bacterial-host interaction rather than affect the host receptor (96). The above work consistently demonstrated that RNPs have non-lethal, non-single-target anti-*H.pylori* adhesion ability, providing a safe window for the development of anti-infective dietary supplements. However, the existing research is still limited to *in vitro* static models, lacking gastric acid-mucus shear, intestinal flora interaction and *in vivo* pharmacokinetic data. In the future, it is necessary to integrate multi-omics, organoid-microorganism co-culture and animal infection models to systematically evaluate their efficacy persistence, dose-effect relationship and synergistic potential with existing antibiotics.

### 5.7 Anti-aging activity

The accumulation of advanced glycation end products (AGEs) is one of the core mechanisms that drive skin aging, vascular sclerosis, and neurodegeneration (97, 98). Finding safe and efficient AGEs inhibitors has become a hot topic in anti-aging research. Recently, RNPs showed a significant blocking effect on the three stages of Amadori products, dicarbonyl intermediates and AGEs in an *in vitro* glycation model. Yu et al. (83) found that BCP inhibited Amadori products, dicarbonyl compounds and AGEs by 34.8 %, 30.8 % and 43.1 %, respectively, in the glucose-bovine serum albumin model, which significantly blocked AGEs-related production and could be used for the development of functional foods or nutritional preparations for anti-aging. Xu et al. (63) found that BCP showed better inhibitory effects on the three pathways (Amadori products, dicarbonyl compounds, AGEs) of protein non-enzymatic glycosylation reaction in the range of 0.05~0.40 mg/mL than the positive control aminoguanidine, and increased with the increase of concentration, especially for the middle and late products. The above studies have consistently shown that RNPs have the potential to be developed as anti-aging functional foods or nutritional preparations. However, the existing data are limited to *in vitro* static models and lack of verification of skin cells, tissues and even *in vivo* levels. In the future, it is necessary to combine the reconstruction of human epidermis, animal models and structure-function studies to clarify its bioavailability, long-term safety and real anti-aging efficacy.

### 5.8 Other bioactivities

In addition, *R. nigrum*-related polysaccharides have also been reported to possess multiple biological activities, including anti-gout effects, skin repair properties, and potential relevance to Alzheimer's disease (AD) research. The heteropolysaccharides PRNP-1 and PRNP-2 isolated by Huang et al. (58) showed reversible competitive inhibition on XOD. Fluorescence quenching and molecular docking showed that the hydrogen bond network formed by PRNP-2 and XOD was more stable. In hyperuricemia mice, PRNPs significantly reduced serum uric acid (UA) and creatinine (Cr), showing the potential of natural low-toxic XOD inhibitors, providing a new treatment for gout and hyperuricemia. The high molecular weight arabinogalactan protein F2 obtained from blackcurrant seeds by Zippel et al. (71) has no cytotoxicity, but can increase the dehydrogenase activity and proliferation rate of fibroblasts and keratinocytes. Gene expression analysis showed that F2 up-regulated DNA repair, ECM protein and signal transduction-related genes, down-regulated some cytochrome P450 metabolic genes, and could be internalized through the cytoskeleton-dependent endoplasmic reticulum pathway, providing metabolic and genetic evidence for skin regeneration. It provides a scientific basis for the development of skin repair and regeneration products based on natural plant polysaccharides. *In vitro* experiments have shown that the inhibition rates of blackcurrant polysaccharides PRTP and PRBP on acetylcholinesterase (AChE) at 5 mg/mL were 30.13 % and 34.61 %, respectively, showing a dose-dependent reversible mixed inhibition. Static fluorescence quenching, ITC and molecular docking showed that PRBP preferentially binds to AChE active center and external sites due to higher affinity, which provides a theoretical basis for the development of new AChE inhibitors, and also suggests a potential direction for the adjuvant intervention of neurodegenerative diseases such as Alzheimer's disease (59).

Taken together, the above studies consistently indicate that RNPs show preliminary promise in anti-gout, skin repair, and AD-related research. However, it must be emphasized that the current body of evidence for each of these activities remains limited. For anti-gout activity, only one study has reported *in vivo* efficacy in a hyperuricemia mouse model, and no independent replication or chronic safety data are available. For skin repair, the evidence is confined to *in vitro* cell-based assays, with no *in vivo* wound healing or regeneration models reported to date. For anti-AD potential, the available data are exclusively derived from *in vitro* acetylcholinesterase inhibition assays and molecular docking analyses, lacking any direct *in vivo* cognitive behavioral assessments or validation in AD animal models. Therefore, further well-designed *in vivo* studies, preferably using multiple animal models and standardized polysaccharide preparations, are urgently needed to substantiate these bioactivities and elucidate their underlying mechanisms before RNPs can be genuinely recognized as natural functional factors with multi-indication efficacy.

### 5.9 Interference of co-extracted polyphenols and polysaccharide-polyphenol complexes in bioactivity evaluation

The fruits, leaves, and processing by-products of blackcurrant are known to be rich in various phenolic compounds, including anthocyanins, flavonols, phenolic acids, and ellagitannins (35). These phenolic compounds themselves have strong antioxidant, anti-inflammatory, and immunomodulatory activities (35). During conventional water or alcohol extraction, polyphenols and polysaccharides are easily co-extracted. They may form polyphenol-polysaccharide complexes through non-covalent interactions or covalent bonds (99). This phenomenon has been confirmed in blackcurrant and its processing residues. For example, Kosmala et al. (100) found that after extracting polyphenols with ethanol or acetone, the sugar composition and hydration properties of alcohol-insoluble solids from blackcurrant pomace changed significantly. This suggests the existence of physical or chemical binding between polyphenols and cell wall polysaccharides. Bu et al. (101) achieved simultaneous extraction of blackcurrant polysaccharides and phenolic compounds using a microwave-assisted ethanol/ammonium sulfate aqueous two-phase system. This further demonstrated the coexistence and separation possibility of the two components during extraction.

However, most current studies on the biological activities of RNPs (especially antioxidant, anti-inflammatory, and immunomodulatory activities) have only performed deproteinization on crude polysaccharides. Very few have systematically removed or quantitatively characterized co-extracted polyphenols or polyphenol-polysaccharide complexes. Considering the strong antioxidant capacity of blackcurrant polyphenols (35), even a small residual amount of phenolic substances may significantly contribute to the apparent activity of RNPs. They may even produce synergistic or additive effects (99). Therefore, fully attributing the activity of RNPs to the polysaccharide backbone alone currently lacks sufficient experimental evidence. Similar issues have also been noted in other polyphenol-rich plant matrices. Zhao et al. (102) pointed out that ellagitannins and other phenolic compounds in walnut pellicle can interact with proteins and polysaccharides, thus affecting the accurate evaluation of the functional activities of isolated products. This example suggests that for research on RNPs, stricter purification strategies and control experiments are needed to distinguish the true contributions of polysaccharides and polyphenols.

Based on the above analysis, future studies on RNPs should, in addition to routine deproteinization, include steps to remove non-covalently bound polyphenols, such as using macroporous adsorption resin or solid-phase extraction. For potentially covalently bound polyphenols, alkaline hydrolysis or enzymatic treatment can be used to release bound phenolics, followed by qualitative and quantitative analysis using HPLC-MS/MS (99, 100). In addition, the dose-response relationship in different bioactivity assays should be compared. This will help clarify the respective activity weights of the polysaccharide backbone and the bound polyphenols. Secondly, when reporting the biological activities of RNPs, the polyphenol fingerprint of the product before and after purification should be provided simultaneously. The types and contents of residual phenolics should be clearly defined to ensure the attribution of conclusions (35, 101). Finally, we should refer to the comprehensive separation and characterization strategies used for polyphenol-rich plant matrices to establish a standardized “dephenolization-activity validation” system suitable for RNPs (102).

In summary, carefully evaluating the potential interference of co-extracted polyphenols and polyphenol-polysaccharide complexes on the activity of RNPs is an unavoidable scientific issue in current research in this field. Only by strictly removing or quantifying the contribution of phenolics can the true biological activity of the RNPs polysaccharide backbone be reflected. This will provide a reliable theoretical basis for the subsequent development of functional foods and pharmaceuticals.

## 6 Structure-activity relationships

**Figure 5:**
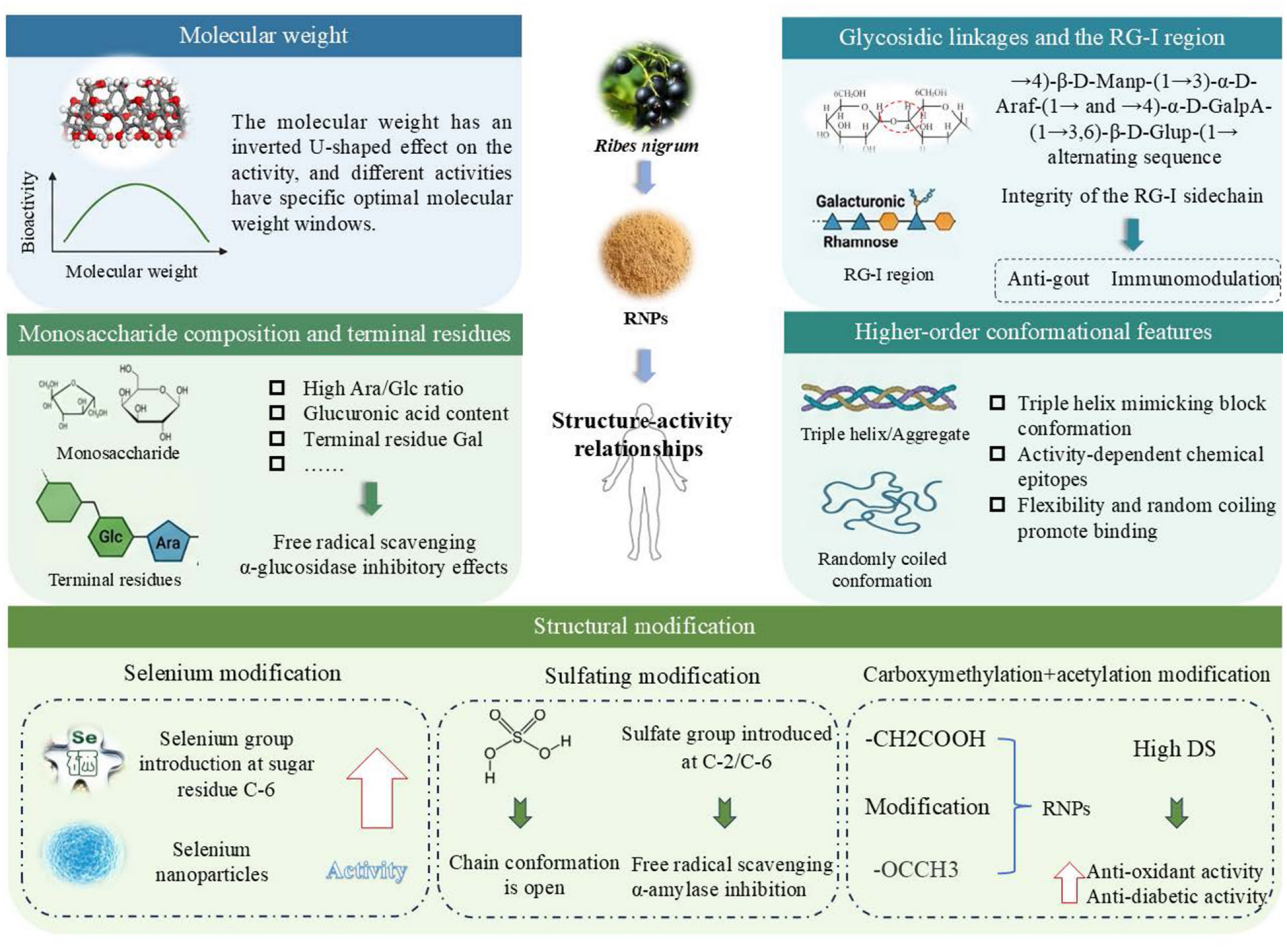
Schematic representation of the structure-activity relationship of RNPs.

The study of structure-activity relationship of polysaccharides is the core link to reveal its biological activity mechanism and realize the directional function design. A large number of studies have shown that the biological activity of polysaccharides is not determined by a single factor, but the result of their own structural characteristics and external structural modification (103). Systematic analysis of the structural characteristics of polysaccharides and the influence mechanism of structural modification strategies on their biological activities not only helps to deeply understand their structure-activity relationship, but also provides a theoretical basis and technical path for the development of efficient and targeted polysaccharide functional products (104). To this end, this study systematically analyzed and mapped the structure-activity relationship of RNPs, and the relevant core information was shown in Figure 5.

### 6.1 Molecular weight

It was found that the activity of RNPs was closely related to the molecular weight. Xu et al. (41) degraded BCP (3.26 × 10^4^ kDa) to DBCP-1 (1.30 × 10^4^ kDa) and DBCP-2 (9.62 × 10^3^ kDa) by Fe^2+^-H_2_O_2_. After the molecular weight decreased, the specific surface area increased and the water solubility increased. The DPPH free radical, hydroxyl radical, superoxide anion radical scavenging, reducing power and metal chelating ability of DBCP-2 exceeded the original sugar, and the inhibition of α-amylase and α-glucosidase was significantly improved. Yang et al. (50) further confirmed that the lowest molecular weight component U-700 obtained by ultrasonic degradation had the best effect in inhibiting erythrocyte hemolysis, increasing GSH and reducing MDA, and directly locking the active site exposure after the molecular weight decreased to further enhance the anti-oxidant activity of polysaccharides. In addition, the comparison of high and low molecular weight components obtained by ethanol fractionation showed that low molecular weight CAPS-l.m. was much higher than ultra-high molecular weight CAPS-h.m. in macrophage activation and IL-12p70 secretion, suggesting that moderate chain length and high water solubility are the premise of immune receptor recognition (64). However, *in vivo* anti-tumor experiments of pectin-type CAPS found that the activity peak of β-galactosidase appeared when the 230 kDa main chain was cut into 19 kDa, and the activity was completely lost when it continued to degrade to 2.4 kDa. It was proved that the activity required continuous oligomeric pectin segments of ≥ 10 kDa and < 50 kDa to retain the negative charge array necessary for TLR4 recognition (89). *R. nigrum* seed acidic arabinogalactan showed *Helicobacter pylori* adhesion blocking activity in the high molecular weight range of 1072/339 kDa, and the water washing fractions with low molecular weight and low charge density were completely inactivated. It was further found that only high branching degree+acidic uronic acid substitution+sufficient chain length can form a multivalent anion interface and competitively occupy the bacterial adhesin site (96).

These results indicate that the effect of molecular weight on the antagonistic activity is inverted U-shaped, and there are different optimal windows for different activities, which need to be considered in combination with receptor recognition mode and *in vivo* barrier penetration requirements. In the future, a systematic molecular weight fractionation library should be established. Combined with *in vitro* and *in vivo* activity evaluations, a precise molecular weight–activity relationship map for different bioactivities can be constructed. Furthermore, the cascading effects of molecular weight variations on the solution conformation of polysaccharides, receptor binding modes, and *in vivo* pharmacokinetic behavior should be thoroughly investigated. This will provide a scientific basis for the targeted preparation and formulation design of highly active RNPs through precise regulation of molecular weight.

### 6.2 Monosaccharide composition and terminal residues

Monosaccharide composition and terminal residues also significantly affect the biological activity of polysaccharides. BCP I has inhibitory activity on α-amylase, which is closely related to its high Ara/Glc ratio, moderate molecular weight and rich polar groups (85). In addition, PRNP-2 has a negative zeta potential due to high uronic acid, and its aqueous dispersion and thermal stability are better than PRNP-1. It is easier to form tight complexes with His251, Lys270, Tyr392, and Arg1330 of XOD by hydrogen bond/hydrophobic interaction (58). Ultrasound treatment also increased the exposure of reducing sugar and uronic acid, increased negative charge, increased hydrogen supply capacity and metal chelating sites, and enhanced free radical scavenging and α-glucosidase inhibition (62). The high molecular weight arabinogalactan protein F2 was completely inactivated after removing the terminal Gal, indicating that the activity depends on the fine epitope of terminal galactose, rather than molecular weight or triple helix (71).

These results indicate that uronic acid content is an important charge switch for RNPs activity, and specific monosaccharides or terminal residues directly determine polysaccharide activity. In the future, techniques such as gene knockout, chemical modification, and surface plasmon resonance should be further employed to precisely elucidate the binding modes and affinity constants between specific monosaccharide residues and target proteins. Concurrently, activity prediction models based on monosaccharide fingerprinting can be developed to guide the targeted screening and preparation of highly active RNPs. Furthermore, the impact of terminal residue modifications on *in vivo* activity should be validated at both the cellular and animal levels to confirm their general applicability as functional epitopes.

### 6.3 Glycosidic linkages and the RG-I region

In addition, glycosidic bonds also have an inseparable effect on biological activity. The → 4)-β-D-Manp-(1 → 3)-α-D-Araf-(1 → and → 4)-α-D-GalpA-(1 → 3,6)-β-D-Glup-(1 → alternating sequence in the main chain of PRNP-2 produces a rigid-flexible block conformation, so that the Araf-GalpA fragment can be deeply inserted into the XOD catalytic channel and induce the conversion of α-helix to β-sheet, thereby efficiently inhibiting XOD and exerting anti-gout activity (58). It is noteworthy that the RG-I region—composed of alternating α-1,2-linked rhamnose and α-1,4-linked galacturonic acid residues, with neutral side chains such as arabinans and galactans attached at the O-4 position of rhamnose—is closely associated with the immunomodulatory activity of pectin-type polysaccharides (53, 58, 72). Different extraction methods significantly affect the integrity of RG-I in RNPs, and differences in structural preservation are directly reflected in their biological activity. The ability of PRSP-1 to induce TNF-α production in macrophages is markedly stronger than that of BCP, demonstrating that the integrity of RG-I side chains serves as an important structural basis for immunoenhancing activity (53). Furthermore, specific modifications of side chains are also critical. Removal of terminal galactose from the blackcurrant seed arabinogalactan protein F2 completely abolishes its anti-adhesion activity against Helicobacter pylori, indicating that terminal galactose is a fine epitope essential for its activity (72, 96). Conversely, excessive debranching (e.g., non-specific enzymatic cleavage in mixed enzyme extraction) significantly reduces the immunomodulatory or anti-inflammatory activity of polysaccharides (64, 89). For CAPS, which relies on TLR4 recognition, the retention of side chains with appropriate length and charge density is a prerequisite for activating the MyD88 signaling pathway (47).

In the future, a quantitative relationship model for glycosidic bond configuration - side chain structure - biological activity should be systematically established. Combined with enzymatic directed modification and structural characterization techniques, the minimal active epitope of the RG-I region should be precisely analyzed. Additionally, the protective strategies for the integrity of RG-I by different extraction methods need to be deeply explored, in order to achieve the targeted preparation of highly active RNPs and their precise application in the fields of immunomodulation and anti-infection.

### 6.4 Higher-order conformational features

In addition to molecular weight and monosaccharide composition, the higher-order conformational characteristics of polysaccharides—such as triple helix structure, chain flexibility, and random coil—also play critical roles in their biological activities (105). Although there is currently no direct evidence for a canonical triple helix structure in RNPs, the rigid-flexible block conformation arising from the alternating sequences of → 4)-β-D-Manp-(1 → 3)-α-D-Araf-(1 → and → 4)-α-D-GalpA-(1 → 3,6)-β-D-Glup-(1 → in PRNP-2 may mimic certain functional features of a triple helix (58). Notably, not all RNPs depend on high molecular weight aggregates or triple helix conformations for their activity. Zippel et al. (71) isolated a high molecular weight arabinogalactan protein, F2, from blackcurrant seeds. After depolymerization under chaotropic conditions, the resulting low-molecular-weight subfractions showed no significant difference from unfractionated F2 in stimulating dehydrogenase activity in skin fibroblasts and keratinocytes. This finding indicates that the cellular activity of F2 is not dependent on its higher-order aggregated state, but rather on specific sugar residue compositions or glycosidic linkage patterns. This counterexample suggests that the structure-activity relationships of blackcurrant polysaccharides are diverse: some fractions may rely on chain flexibility and charge distribution, whereas others depend more on specific chemical structural epitopes rather than supramolecular aggregation. Chain flexibility also influences the interaction of RNPs with enzymes and receptors. Studies have revealed that RNPs possess a certain degree of flexibility. This balanced flexibility may facilitate the insertion of Araf-GalpA segments into the catalytic pocket of XOD, as suggested by molecular docking studies (58). With respect to random coil conformation, dynamic light scattering and rheological data indicate that most RNPs exist as random coils in aqueous solution, which may enhance their accessibility to membrane receptors (50, 106).

In summary, higher-order conformational features exert nuanced and context-dependent effects on the biological activities of RNPs. Future studies should employ methods such as Congo red staining, circular dichroism spectroscopy, atomic force microscopy, and computational modeling to systematically dissect the conformational determinants underlying the bioactivities of RNPs.

### 6.5 Structural modification

Although natural polysaccharides have a variety of biological activities, their poor water solubility, weak targeting, and limited activity intensity often limit their practical application. In recent years, researchers have found that artificial structural modification can modify the structure of polysaccharides while retaining the advantages of the parent skeleton, thereby significantly amplifying or endowing them with new functions (107). Zhao et al. (53) found that the molecular weight of RNPs was significantly reduced after selenization modification, and the water solubility, physical stability and rheological properties were significantly improved. These changes in physical and chemical properties provided favorable conditions for its absorption and utilization *in vivo*. FT-IR and NMR analysis confirmed that the selenium group was mainly introduced to the C-6 position of the sugar residue. This structural modification not only retained the original glycosidic bond connection mode, but also enhanced the interaction between the polysaccharide and the target enzyme by reducing the particle size, increasing the specific surface area and negative potential. At the same time, the activity intensity of the modified polysaccharide was significantly higher than that of the natural polysaccharide, indicating that the selenization modification not only improved the physical and chemical properties of the RNPs, but also enhanced its molecular recognition and binding stability with digestive enzymes through fine chemical modification, thereby significantly improving its anti-diabetic activity. Other scholars have prepared stable selenium nanoparticle polysaccharides (RP-SeNPs). Compared with single selenium nanoparticles (SeNPs), RP-SeNPs exhibit smaller particle size, narrower particle size distribution, higher negative charge and better stability. These properties make RP-SeNPs significantly superior to SeNPs in terms of anti-glycation and α-glucosidase inhibitory activity (80).

In addition, Xu et al. (54) revealed that with the increase of DS from 0.53 to 1.28, the molecular weight of RNPs sulfated derivatives (SBCPs) increased slightly, Mw/Mn widened, and the sulfate group was mainly connected to C-2 and C-6 positions, which promoted the molar ratio of monosaccharides from rhamnose-glucose to arabinose-galactose, and the chain conformation was more open. At the same time, this structural change synergistically enhanced the interaction between hydrogen donor and electrostatic interaction, so that SBCP-1 with high DS was significantly superior to low DS samples and original BCP in DPPH, hydroxyl and superoxide anion scavenging, reducing power, Fe^2+^ chelation and other anti-oxidant indexes, as well as α-amylase inhibitory activity. Duan et al. (18) systematically compared the relationship between structural characteristics and anti-oxidant activity of *R. nigrum* carboxymethylated polysaccharides (CRNPs) with different DS and their natural precursors (RNP). On the basis of maintaining the original monosaccharide composition, the molecular weight, water solubility and carboxyl content of CRNPs increased with the increase of DS, indicating that the introduction of carboxymethyl groups enhanced the molecular polarity and structural stability. In addition, carboxymethylation enhanced its anti-oxidant activity, including free radical scavenging ability, lipid peroxidation inhibition and red blood cell oxidative damage protection, and the activity intensity was positively correlated with DS. Other scholars found that acetylation modification significantly reshaped the molar ratio of monosaccharides of RNPs. At the same time, the scavenging ability of free radicals and the inhibitory activity of α-amylase and α-glucosidase after acetylation were enhanced in a DS-dependent manner. Enzyme kinetics revealed that the reversible competitive inhibition of acetylated products on α-glucosidase was enhanced with the increase of DS, and the Ki value decreased synchronously (43).

In summary, artificial structural modification can accurately regulate the physical and chemical properties of natural polysaccharides, and the improvement of biological activity is often related to the type and density of modification, which provides a solid theoretical basis for promoting the practical application of polysaccharides. However, most of the current studies focus on specific polysaccharides and single modification effects, and systematic comparison of composite modification strategies. In addition, the intrinsic mechanism between structural changes and *in vivo* biological activity has not been fully elucidated, which may hinder the transformation of modification technology into clinical or industrial applications.

## 7 Safety evaluation

RNPs are considered as non-toxic templates due to their high dietary exposure history, but there are few studies on the dose-toxicity relationship, functional group-toxicity coupling and host-microorganism co-metabolism in the literature. Huang et al. (58) systematically evaluated the *in vitro* biocompatibility of PRNP-1 and PRNP-2 polysaccharides on human renal tubular epithelial HK-2 cells. In HK-2 renal cells, PRNP-1 and PRNP-2 maintained >85 % viability at up to 5 mg/mL, which is above the ISO 10993-5 cytotoxicity threshold (< 70 %). Nevertheless, this single-cell model does not capture systemic toxicity, and *in vivo* safety evaluations remain necessary before translational applications. Some scholars have evaluated the cytotoxicity of polysaccharides (CAPS) on bone marrow-derived dendritic cells (BMDCs) through cell experiments. After co-cultured with 10 or 100 μg/mL CAPS for 24 h, BMDCs showed no cytotoxicity, cell viability was not significantly affected, and had good biocompatibility (47). Zippel et al. (71) evaluated the safety of the arabinogalactan protein F2 (derived from *R. nigrum* seeds) on primary human dermal fibroblasts and HaCaT keratinocytes using the lactate dehydrogenase (LDH) release assay. At concentrations of 10 and 100 μg/mL, no significant LDH release was detected, indicating that F2 did not compromise cell membrane integrity or induce necrosis. However, it should be noted that the LDH assay primarily assesses membrane damage and does not capture potential metabolic or genotoxic effects. Complementary assays are warranted in future studies to establish a comprehensive safety profile. Nevertheless, the available evidence, including enhanced dehydrogenase activity and downregulation of cytochrome P450 isoforms as observed in gene expression profiling, collectively supports the safety of F2 within the tested concentration range. The *in vitro* WST-1 experiment showed that the direct cytotoxicity of *R. nigrum* polysaccharides (CAPS) on Ehrlich ascites tumor cells was weak, and the IC_50_ value was as high as 760 μg/mL, which was much higher than the blood concentration reached after oral administration, suggesting that it had extremely low toxicity to normal tissues within the effective dose range and had an excellent safety window (64). In addition, *R. nigrum* arabinogalactan protein F2 had no cytotoxicity to Helicobacter pylori and AGS gastric epithelial cells in the range of 0.5~4 mg/mL, and did not induce up-regulation of bacterial virulence gene expression, suggesting that it was safe (95).

These studies have shown that RNPs have a wide safety window under acute and low-dose conditions, with no overt cytotoxicity or adverse effects reported. However, current evidence remains largely confined to *in vitro* single-cell-layer models or short-term animal studies, leaving several critical gaps: the effects of high-dose, long-term exposure, the potential accumulation of chemically modified RNPs, and the possible occurrence of class-specific adverse reactions such as intestinal gas production, diarrhea, or excessive immune activation—none of which have been observed for RNPs to date but are known for other fermentable or immunostimulatory polysaccharides. Therefore, future *in vivo* toxicology studies should systematically compare the LD50 and maximum tolerated dose of native, selenized, sulfated, and carboxymethylated RNPs, while also monitoring gastrointestinal tolerability and immune activation markers to define a true no-observed-adverse-effect level and ensure the safe application of RNPs.

## 8 Related bibliometrics of RNPs

RNPs have become a hot topic in the research of functional foods and natural medicines due to their anti-oxidant and immunomodulatory activities. The number of related literatures has grown rapidly, and it is urgent to systematically sort out their research context, hot topics and evolution trends through bibliometric methods (108). To this end, this study uses the bibliometric paradigm for the first time to conduct a full-dimensional scan of RNPs research papers included in the Web of Science core collection and Pubmed database, and reveals the knowledge evolution path through the co-word network and cooperation map, in order to provide data-driven decision-making reference for subsequent scientific research layout and industrial application.

**Figure 6:**
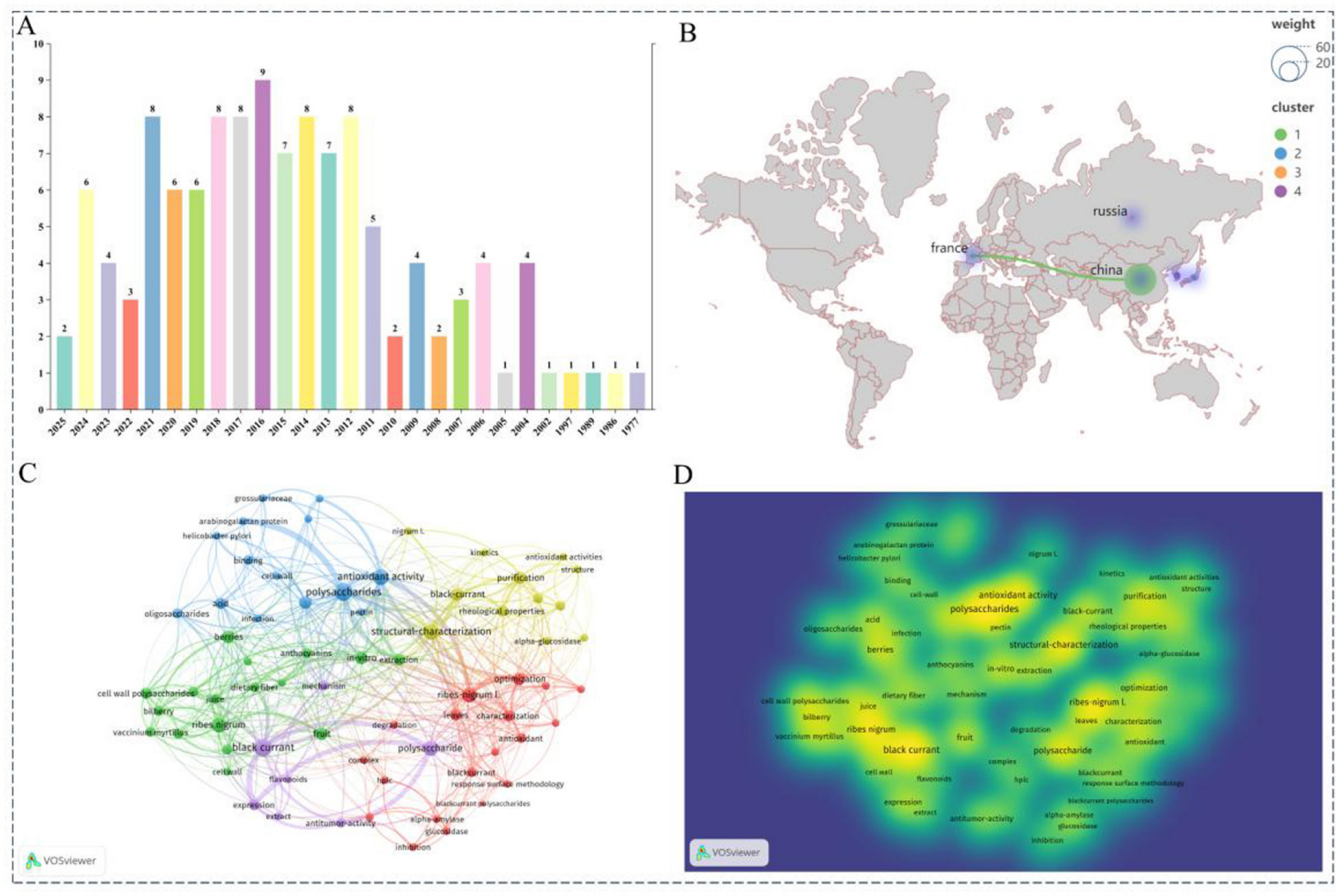
Bibliometric analysis of RNPs-related literature: **(A)** Publication trend of RNPs-related literature, **(B)** Publication countries and their collaboration networks, **(C, D)** Keyword analysis of RNPs-related studies.

Through bibliometric analysis of literature published from 1977 to 2025, research on RNPs has grown steadily since 2005, with annual publications reaching a maximum of 9 in 2016 and ranging between 3~7 per year from 2017 to 2021. These absolute numbers remain low, indicating a relatively small but specialized niche. Normalized against all polysaccharide-focused articles in Web of Science over the same period, RNPs publications consistently accounted for only 0.005~0.01 % annually, confirming a stable subfield without pronounced fluctuations. The slight decline after 2021 can be attributed to: (1) a natural shift from routine extraction and activity screening toward in-depth mechanistic studies and structurally defined homogeneous polysaccharides, which require longer research cycles; (2) the global impact of the COVID-19 pandemic on non-urgent research; and (3) partial diversion of attention to emerging topics such as gut microbiota modulation and nano-delivery systems. Collectively, the bibliometric evidence suggests that the RNPs field has entered a mature consolidation phase, where future advances will depend on mechanistic breakthroughs, clinical translation, and international collaboration rather than continued growth in publication volume (Figure 6A). In terms of national literature output and influence, China demonstrates the highest productivity and academic influence. Based on the bibliometric analysis of the Web of Science Core Collection and PubMed database, China has published 17 papers in the field of RNPs research, with a total citation count of 1,071 and an h-index of 11, ranking first among all countries. In contrast, the second-largest producer, the Netherlands, has published 4 papers, with a total citation count of 207 and an h-index of 4. These data objectively confirm the conclusion that China is the core output country for RNPs research. From the perspective of the international cooperation network, there are obvious cooperative links between China and France, indicating stable and significant international cooperation in this field (Figure 6B). It should be noted that the design of Figure 6B is to display the main publishing countries and their partnerships, and therefore does not include specific bibliometric data. By analyzing the keywords in the RNPs-related research knowledge domain, it can be seen that ‘polysaccharides' and ‘anti-oxidant activity' are at the core of the research density, indicating that the exploration of polysaccharides and their anti-oxidant properties is a field of concern. At the same time, keywords such as “black currant,” “anthocyanins,” and “flavonoids” also showed a high research density, highlighting the attention to phytochemicals in black currant. In addition, terms such as “structural-characterization,” “optimization,” and “response surface methodology” also have a considerable density distribution, indicating that structural analysis of active substances and optimization of extraction or preparation processes are also important components in this field (Figures 6C, D). In summary, these two maps reveal that the research in this field takes the bioactive components of RNPs as the core, and integrates the research on its characteristics, structure and related technical methods. Future research can further explore a variety of biological activities and mechanisms of RNPs in addition to anti-oxidant properties, and use advanced technology to accurately characterize the structure of active substances. To further expand the application of RNPs in food, medicine and cosmetics, evaluate its safety and effectiveness, and provide reference for practical transformation.

## 9 Potential applications

**Figure 7:**
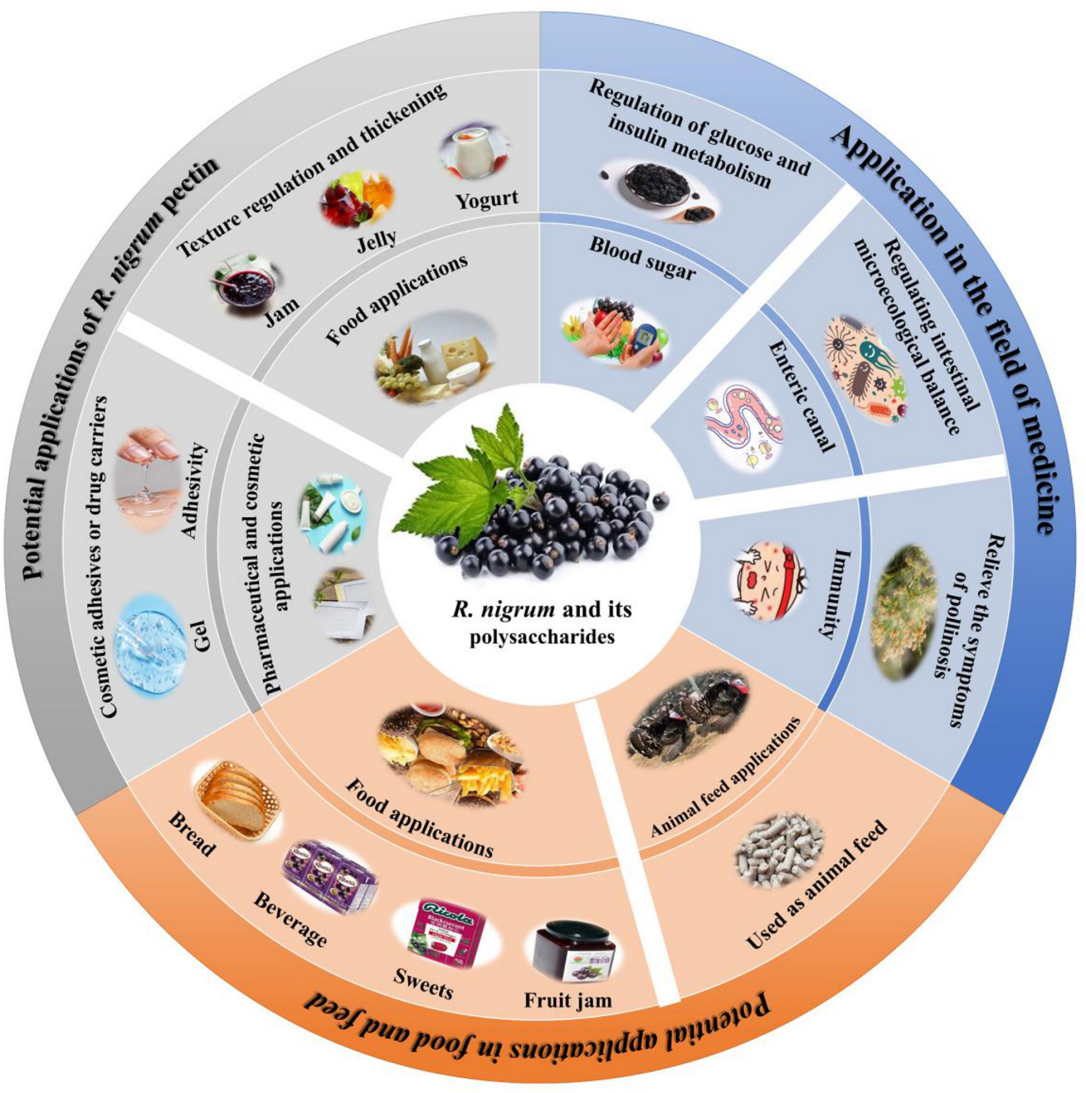
Visual summary of the potential application directions of RNPs.

*R. nigrum*, its fruit has attracted much attention in recent years due to its rich nutritional value and potential health benefits (109). Studies have shown that blackcurrant polysaccharides not only have significant anti-oxidant, immunomodulatory and anti-inflammatory effects, but also show good prospects in improving intestinal microecology and regulating glycolipid metabolism. With the increasing demand for functional foods and natural medicines, the application research of RNPs is gradually becoming a hot direction in the field of food, medicine and health care products. Figure 7 further illustrates the potential application directions of *R. nigrum* and RNPs.

Pectin is a linear polysaccharide composed of multiple structural units. Scholars have found that *Ribes* pectin possesses excellent physicochemical and functional properties, showing broad application potential in food, medicine, and cosmetics. Its high methoxyl pectin can form a stable gel under acidic conditions and has good rheological properties. It is not only suitable for texture regulation and thickening in food such as jam, jelly and yogurt, but also can be used as a cosmetic adhesive or drug carrier (42). Michał et al. (106) revealed the molecular behavior and relaxation phenomenon of *R. nigrum* pectin in aqueous solution. This pectin shows a high friction radius and a low overlap concentration, and in dilute solution, it exhibits strong intermolecular interaction and weak hydration capacity. Both NMR and osmotic pressure results show that its interchain aggregation tendency is significant. With the increase in temperature, the chain flexibility decreases, and the hydration capacity weakens, resulting in a decrease in viscosity and an extension of relaxation time. Dynamic light scattering and rheological analysis further show that this pectin can form a weak gel structure at a relatively high concentration, exhibiting significant shear-thinning behavior and viscoelastic characteristics. It has good thickening and structure regulation potential in food systems and is suitable for constructing low-concentration gel networks and controlled-release systems.

Beyond the food sector, *R. nigrum* and its polysaccharides also demonstrate extensive application potential and significant research value in the pharmaceutical field. In terms of blood glucose and insulin metabolism regulation, Törrönen et al. (110) conducted a randomized crossover postprandial trial and found that co-consuming a mixed berry puree containing blackcurrant along with other berries (such as bilberries, lingonberries, and cranberries) with an equivalent amount of sucrose significantly suppressed the peaks of blood glucose and insulin in healthy subjects at 15 min postprandial, delayed and prolonged the blood glucose rebound process after 90 min, and doubled the glycemic curve profile index of capillary and venous blood glucose. This suggests that the whole berry mixture can improve short-term blood glucose homeostasis after sucrose loading and reduce the risk of hyperinsulinemia by slowing down the rates of sucrose digestion and glucose absorption (110). However, it is important to note that this study used a complex mixture of whole berries rather than isolated RNPs. Therefore, the observed effects cannot be directly attributed to RNPs alone. Nevertheless, these findings provide a strong rationale for future dedicated human trials using purified RNPs or standardized blackcurrant polysaccharide extracts. Another randomized crossover trial involving healthy women further verified that, compared with consuming sucrose alone, intake of 150 g of whole blackcurrants or 300 mL of blackcurrant nectar significantly inhibited the peaks of blood glucose and insulin at 15 min postprandial, delayed the glucose absorption process within 60~120 min, and simultaneously prevented late hypoglycemia and free fatty acid rebound. These results demonstrate the benefits of whole blackcurrant fruits, but do not provide direct evidence for RNPs alone. These findings confirm the feasibility of blackcurrant as a whole food for regulating postprandial blood glucose homeostasis (111). Relevant results from this study also showed that when blackcurrant was consumed as part of a mixed berry blend, it significantly reduced the postprandial insulin response in healthy women. After adding this mixed berry blend to meals with white bread or rye bread as the carbohydrate source, the insulin area under the curve (AUC) decreased by 39 % and 40 %, respectively, and the maximum insulin increase was reduced by 22 % and 23 %, respectively. This indicates that the mixed berry blend exerts a synergistic effect in regulating postprandial insulin metabolism, suggesting its potential application value as a functional food ingredient in reducing postprandial insulin demand for high-carbohydrate meals—particularly suitable for dietary intervention strategies aimed at improving insulin sensitivity and regulating metabolic health (112). These effects should be attributed to the entire berry mixture rather than the individual RNP. Further research is needed to assess the specific contribution of RNP.

In the field of intestinal health and disease prevention, studies have confirmed that continuous intake of blackcurrant extract-based products for two weeks significantly increased the counts of beneficial bacteria in feces, reduced the abundance of potential pathogenic bacteria, significantly inhibited the activity of bacterial β-glucuronidase, and decreased fecal pH. These results demonstrate that blackcurrant extract can exert potential chemopreventive effects against colon cancer by regulating intestinal microecological balance, providing a scientific basis for the development of blackcurrant-based functional foods or dietary intervention protocols (113). In the field of immunomodulation and anti-allergy, a randomized, double-blind, placebo-controlled trial evaluated the intervention effect of blackcurrant-derived polysaccharides (CAPS) on patients with Japanese cedar pollinosis. The results showed that although CAPS did not significantly improve the daily symptom score, it significantly inhibited the progression of inferior turbinate mucosal swelling in terms of objective indicators, and alleviated the worsening trend of ocular symptoms in the quality-of-life questionnaire—suggesting its potential benefits in relieving allergic rhinoconjunctivitis. Mechanistic analysis indicated that CAPS may exert anti-allergic effects by regulating the expression of immune-related genes, inhibiting IgE production, and suppressing Th2-type cytokines. This study is the first to confirm in human trials that blackcurrant polysaccharides, as a food supplement, can be safely used to assist in alleviating pollinosis symptoms, with clear application prospects particularly in improving ocular allergic manifestations (114).

In the field of food, *R. nigrum* and its polysaccharides are widely used. Its juice powder, frozen fruit, extract and polysaccharides are widely used in beverages, baking, dairy products, candies, jams and other functional foods (115–117). It not only gives the product natural color and unique flavor, but also significantly improves the nutritional value and health attributes because it is rich in active ingredients such as anthocyanins, vitamin C and polysaccharides (95, 118). In addition to the field of food and medicine, blackcurrant and its related by-products also have application value in the field of feed. As a by-product of blackcurrant processing, blackcurrant pomace is rich in functional components such as non-starch polysaccharides (such as cellulose), polyphenols and pectin, and is a potential high-quality feed additive. Studies have shown that adding 5 % blackcurrant pomace to the turkey diet can significantly improve the fermentation pattern and microecological function of the hindgut of turkey. On the one hand, it can significantly reduce the dry matter concentration of small intestinal chyme and increase the activity of beneficial enzymes in the hindgut. On the other hand, it can reduce the production of propionic acid, increase the content of butyric acid, and reduce the concentration of spoilage branched-chain fatty acids and ammonia by selectively adjusting the proportion of hindgut fermentation products. The above results confirm that blackcurrant pomace can promote the transformation of animal intestinal microorganisms to beneficial intestinal health by regulating the metabolic direction of animal intestinal microorganisms, and has the feasibility and application potential as an animal feed additive (119).

## 10 Conclusion and future prospects

In recent years, RNPs have emerged as a class of natural macromolecules. They possess diverse structures and exhibit a wide range of biological activities. This review systematically summarizes recent advances in several areas, including the extraction, purification, structural characterization, biological activities and safety evaluation of RNPs. Overall, RNPs exhibit significant biological activities. For example, they demonstrate antioxidant, anti-diabetic, immunomodulatory, anti-inflammatory, anti-tumor, anti-aging and anti-adhesion activities. These properties make RNPs promising candidates for functional foods, nutraceuticals and drug development.

Despite these promising findings, several critical challenges remain that must be addressed to facilitate the translation of RNPs from laboratory research to industrial and clinical applications:

(1) Standardization of extraction and structural integrity reporting. Future studies on RNPs should systematically report extraction parameters (ultrasonic intensity, pH, temperature, enzyme specificity, duration, etc.) along with key structural characterization data, degree of branching, and evidence of RG-I region integrity. The establishment of a standardized report would facilitate cross-study comparisons and guide researchers in selecting the most appropriate extraction protocols for specific bioactivities. Moreover, RG-I integrity should be recommended as a critical quality control indicator for pectin-type polysaccharides. In addition, standardized and scalable extraction and purification processes are still lacking. Future efforts should focus on developing green, efficient, and low-cost technologies to improve the yield, reproducibility, and structural integrity of RNPs.

(2) Research on the interaction between RNPs and polyphenols. Future studies should first focus on establishing a standardized “dephenolization-activity validation” system. This system should efficiently remove non-covalently bound polyphenols. For covalently bound polyphenols, alkaline hydrolysis or enzymatic treatment should be used to release them, followed by qualitative and quantitative analysis. Secondly, multiple control groups should be set up to systematically compare the dose-response relationships in various bioactivity assays, including antioxidant, anti-inflammatory, and immunomodulatory activities. This will help clarify the respective activity contributions of the polysaccharide backbone and the bound polyphenols. Meanwhile, when reporting the biological activities of RNPs, the polyphenol fingerprints of the products before and after purification should be provided simultaneously. This ensures the attributability and reproducibility of the research conclusions. Ultimately, these efforts will truly reveal the intrinsic structure-activity relationship of the RNPs polysaccharide backbone, providing a reliable scientific basis for their precise application in functional foods and pharmaceuticals.

(3) Research on RNPs as regulatory factors for modulating the intestinal immune axis. Given the poor absorption capacity of high-molecular-weight polysaccharides, future studies should systematically utilize *in vitro* fermentation models, germ-free animal research, and human intervention trials to systematically explore the probiotic effects of structurally defined RNPs. Combining artificial intelligence-assisted metagenomic analysis, targeted drug delivery systems, and metabolite-based biomarkers, a causal relationship between specific RNPs structures, changes in intestinal microorganisms, the production of short-chain fatty acids, and other activities will be established. This multi-omics and modeling approach will be able to design functional foods based on RNPs that are rationally configured according to an individual's intestinal microbiome.

(4) Elucidation of structure-activity relationships. Knowledge on the structure-activity relationships of RNPs remains limited. This limitation arises mainly from two factors. First, natural polysaccharides have inherent structural complexity and heterogeneity. Second, these features make precise characterization difficult using conventional methods. Preliminary structure-activity relationships studies indicate that several factors significantly influence bioactivity. These factors include molecular weight, monosaccharide composition, glycosidic linkages, and terminal residues. However, the exact molecular mechanisms remain unclear. To overcome these bottlenecks, future research should integrate advanced orthogonal analytical techniques with computational modeling and machine learning. Examples of such techniques are NMR spectroscopy, HPSEC-MALS, and atomic force microscopy. This integration will help to systematically decipher structure-activity relationships and construct robust models.

(5) Systematic evaluation of chemical modification strategies remains imperative. Although certain chemical modifications—such as selenylation, sulfation, carboxymethylation, and acetylation—have demonstrated considerable potential in improving the physicochemical properties and bioactivities of RNPs, several critical knowledge gaps persist. Specifically, systematic comparative studies across different modification strategies are still lacking, and the relationships between complex modifications and *in vivo* biological activity warrant further exploration. Additionally, comprehensive assessments of the long-term stability, safety, and *in vivo* efficacy of these modified polysaccharides are urgently needed.

(6) Systematic studies of *in vivo* pharmacokinetics and pharmacodynamics (PK/PD). Knowledge on the pharmacokinetic behavior of RNPs remains limited. Traditional pharmacokinetic models often fail to describe the unique *in vivo* behavior of polysaccharides. Most current studies are limited to *in vitro* assays or acute animal models. Therefore, comprehensive *in vivo* studies are essential. These studies should include PK/PD profiles, biodistribution, and long-term safety evaluations. Such studies will help validate the therapeutic effects of RNPs and guide clinical dosing. We particularly recommend the development of physiologically based pharmacokinetic models. These models should integrate intestinal transit, gut microbiota metabolism, and potential receptor-mediated clearance.

(7) Improvement of toxicology and safety assessment. Knowledge about the safety characteristics of RNPs is still limited. *In vitro* experiments have shown that RNPs have relatively low cytotoxicity. However, systematic toxicological studies are still insufficient. Especially for chemically modified derivatives, the situation is even more so. To bridge this gap, future research should establish comprehensive safety databases. These databases should cover key parameters such as blood compatibility, immunotoxicity, and long-term toxicity. Additionally, systematic toxicological investigations, particularly those focusing on gastrointestinal tolerance and the risk of immune overactivation at high doses, are currently lacking. Subsequent efforts should establish comprehensive safety features, including a clear minimum observation dose without harmful effects. We also suggest establishing a standardized safety assessment system. Such a system will facilitate obtaining regulatory approval and for human applications.

(8) Promotion of clinical translation and formulation development. RNPs have great potential in functional foods, cosmetics, and pharmaceuticals. Despite this potential, clinical trials are almost nonexistent. Future studies should prioritize human intervention trials. These trials should focus on chronic diseases such as diabetes, allergies, and metabolic syndrome. Concurrently, novel formulation strategies based on RNPs should be actively explored. For example, researchers could use nanoencapsulation or exosome delivery systems. These strategies can enhance bioavailability and achieve targeted delivery.

(9) Establishment of quality control and standardization. The current lack of standardized RNP preparations severely affects two things. One is the reproducibility of research results. The other is the regulatory approval process. Future efforts should focus on establishing systematic quality control benchmarks. These benchmarks should include unified specifications for molecular weight distribution, monosaccharide composition, purity, and bioactivity assays.

In summary, RNPs are a class of natural resources. They have both structural diversity and significant biological activities. Therefore, they hold broad prospects in functional foods, nutraceuticals, and therapeutic agents. However, bridging the gap between current knowledge and practical applications requires a collaborative approach. This approach should integrate food science, pharmacology, toxicology, and clinical medicine. Addressing the above challenges will lay a solid foundation for the rational design, safe use, and eventual commercialization of RNPs. In this way, RNPs can effectively promote human health and prevent chronic diseases.
